# Global Trends of Mean and Inequality in Multidimensional Wellbeing: Analysis of 1.2 Million Individuals From 162 Countries, 2009–2019

**DOI:** 10.3389/fpubh.2022.824960

**Published:** 2022-02-14

**Authors:** Koichiro Shiba, Richard G. Cowden, Natasha Gonzalez, Matthew T. Lee, Tim Lomas, Alden Yuanhong Lai, Tyler J. VanderWeele

**Affiliations:** ^1^Department of Epidemiology, Harvard T.H. Chan School of Public Health, Boston, MA, United States; ^2^Human Flourishing Program, Harvard Institute for Quantitative Social Science, Cambridge, MA, United States; ^3^Independent Researcher, Madrid, Spain; ^4^School of Global Public Health, New York University, New York, NY, United States; ^5^Department of Biostatistics, Harvard T.H. Chan School of Public Health, Boston, MA, United States

**Keywords:** human flourishing, multidimensional wellbeing, life evaluation, inequality, epidemiology

## Abstract

**Introduction:**

Human flourishing is a multidimensional concept characterized by a state of complete wellbeing. However, much of the prior research on wellbeing has principally focused on population averages assessed using a single item of wellbeing. This study examined trends in population averages and inequalities for a multidimensional index of wellbeing and compared emergent patterns with those found for Cantril's ladder, a measure of life satisfaction commonly used as a unidimensional index of wellbeing.

**Methods:**

Data were from the Gallup World Poll from the years 2009 to 2019, a repeated cross-sectional survey of nationally representative samples comprising ~1.2 million individuals from 162 countries. We assessed five domains of flourishing: (1) happiness, (2) health, (3) purpose, (4) character, and (5) social relationships. We used the Gini Index to estimate inequalities in wellbeing within populations. We examined and compared country ranking, global and region-specific trajectories of mean and inequality, and relationships with age for flourishing and Cantril's ladder.

**Results:**

Although all trends were highly correlated across the two metrics of wellbeing, we identified distinct patterns in flourishing concerning geography, time, and age relationships that were not observed for Cantril's ladder. Temporal trends and age relationships were different across domains of flourishing. Evidence of changing inequalities in wellbeing was also found, even when population averages were high or stable over time.

**Conclusion:**

Comprehensive measures of wellbeing are needed to capture the complex and changing patterns of wellbeing both within and across populations.

## Introduction

Humans have had a long-standing interest in identifying, understanding, and cultivating the key ingredients of a *flourishing life*, a multidimensional concept reflecting a state of complete wellbeing in which “all aspects of a person's life are good” (1–3). The importance of flourishing is espoused in principles of international agencies dedicated to improving global health (e.g., World Health Organization's constitution) (4). Notions of flourishing are also interwoven into global initiatives established to promote human development throughout the world. For example, the United Nations' 2030 agenda for global transformation includes *good health and wellbeing* as one of its 17 Sustainable Development Goals (5). Similar priorities are reflected in the initiatives of other international entities (e.g., Templeton World Charity Foundation) invested in promoting wellbeing (6). A better understanding of distributions and determinants of wellbeing will provide valuable insight into progress toward many existing global goals and initiatives.

To this end, there are two main limitations in existing empirical research on wellbeing that need to be addressed to support the promotion of wellbeing as a major public health endeavor. First, most studies focus on a narrowly defined (often unidimensional) conception of wellbeing (2, 7–10). This partial assessment of wellbeing is evident in large-scale multi-nation reports, many of which influence policies, strategic agendas, and resource allocation decisions at local and international levels. For example, the World Happiness Report provides an annual country-level comparison of subjective wellbeing (11). This report relies primarily on Cantril's ladder, a single-item measure of self-reported life satisfaction (sometimes referred to as life evaluation) (12). Life satisfaction is commonly used as a measure of unidimensional wellbeing and has some desirable properties (e.g., its conceptual clarity and comparability across studies) (13). Despite the value and potential impact of such analyses, such evidence provides an incomplete picture of wellbeing, because life satisfaction represents only one of a broader set of indicators for human flourishing. A more holistic approach to assessing wellbeing could identify areas of human life that have been over- or underemphasized in policies and agendas, informing future public health priorities (14).

Second, much of the existing research on wellbeing has overlooked the distribution of wellbeing within populations in favor of assessing trends in population averages. An improved understanding of changing inequalities in wellbeing could contribute to policy refinement and more informed resource allocation decisions, which may help address social inequities within and across countries. In existing research on wellbeing inequalities, the focus has usually been on specific aspects of wellbeing (e.g., health and life satisfaction) (15, 16). To date, no study has conducted a systematic global analysis of changing population distributions of wellbeing based on a more comprehensive measure.

Using a dataset with nationally representative sampling consisting of more than one million individuals from 162 countries, the purpose of this study is to assess global and region-specific trajectories of means and inequalities in multidimensional wellbeing (assessed by the items that were available in the data and best capture the multiple domains of wellbeing) from 2009 to 2019 and compare the trends in distributions of wellbeing with more a commonly-used metric (i.e., Cantril's ladder).

## Materials and Methods

### Data

**Figure 1:**
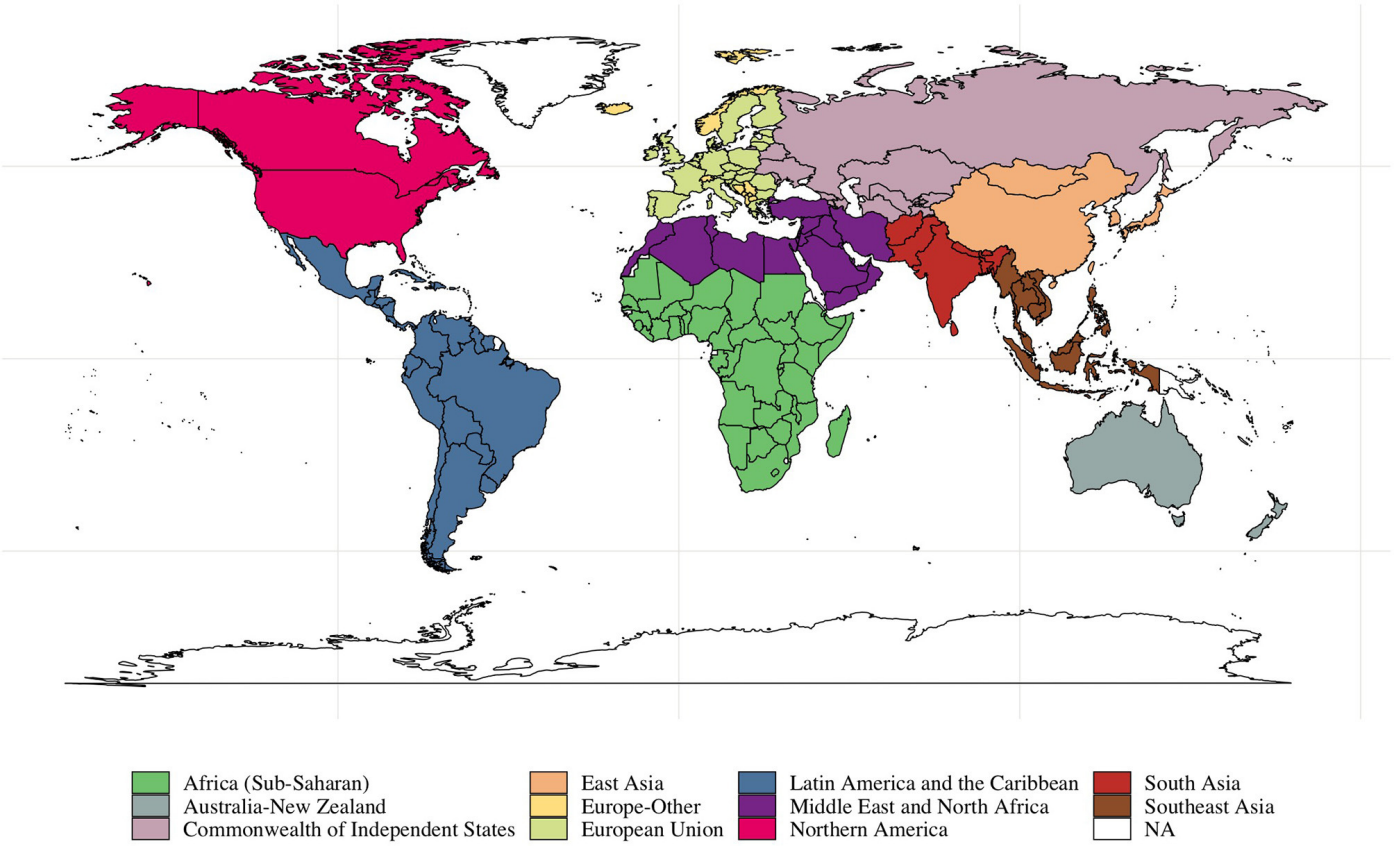
Data availability. Countries are colored by regions. Countries that did not participate in the Gallup World Poll are showed as NA.

Data came from the Gallup World Poll (GWP), a repeated cross-sectional study of nationally representative samples of non-institutionalized residents aged 15 years or older from 168 countries (see Figure 1), covering more than 98 percent of the world's population (17). GWP began in 2005 and collected data from ~1,000 respondents per country annually through 2019 (country-year specific sample size: mean = 1,169, SD = 775, min = 500, max = 13,408). Data were collected *via* face-to-face interviews in low-income countries and *via* random-digit-dialing telephone interviews in high-income countries with 80% or higher telephone coverage of the population. For countries with face-to-face interviews, the samples were obtained using multi-stage sampling, in which GWP sampled clusters of households as Primary Sampling Units (PSUs), followed by households within the PSUs, and then members in each household.

This study used data from 2009 to 2019 because some GWP wellbeing items were not measured in prior years. We excluded six countries (i.e., Saudi Arabia, Jordan, Cuba, Guyana, Oman, and the Maldives) where at least one of the wellbeing measures was completely missing throughout the 11-year period. The final study sample consisted of 1,193,134 observations from 162 countries. Because this study involved secondary analysis of de-identified data, its ethical review was exempted.

### Measurement

Using VanderWeele's (2) multidimensional conception of flourishing as a framework, we assessed five domains of wellbeing that are universally desired and ends in themselves: (1) happiness, (2) health, (3) purpose, (4) character, and (5) social relationships (2). Unlike other theorizing and other existing measures of multidimensional wellbeing, this framework suggests that flourishing consists of something more than one's psychological state and thus covers other dimensions of human wellbeing such as character and physical health as well. We selected three items that corresponded to each domain among the items available in the GWP. Specifically, we first selected candidate items based on their theoretical fit with the domains of interest and then examined between-item correlations to choose three items for each domain. Only two items had discrete responses ranging from 0 to 10. All other items were binary. The full list of items is available in Table 1. Between-item correlations are shown in Supplementary Table 1.

**Table 1:** List of items for the composite flourishing score.

**Domain**	**Item**	**Code**
Happiness	(Happiness 1) On which step of the ladder would you say you personally feel you stand at this time?	Original responses (0 (the worst possible life) to 10 (the best possible life) were rescaled to range from 0 to 1.
Happiness	(Happiness 2) Just your best guess, on which step do you think you will stand in the future, say about 5 years from now?	Original responses (0 (the worst possible life) to 10 (the best possible life) were rescaled to range from 0 to 1.
Happiness	(Happiness 3) Did you experience the following feelings during a lot of the day yesterday? How about enjoyment?	Yes = 1 and No = 0
Health	(Health 1) Do you have any health problems that prevent you from doing any of the things people your age normally can do?	No = 1 and Yes = 0
Health	(Health 2) Did you experience the following feelings during a lot of the day yesterday? How about physical pain?	No = 1 and Yes = 0
Health	(Health 3) Now, please think about yesterday, from the morning until the end of the day. Think about where you were, what you were doing, who you were with, and how you felt: Did you feel well-rested yesterday?	Yes = 1 and No = 0
Purpose	(Purpose 1) In (this country), are you satisfied or dissatisfied with… Your freedom to choose what you do with your life?	Yes = 1 and No = 0
Purpose	(Purpose 2) Did you learn or do something interesting yesterday?	Yes = 1 and No = 0
Purpose	(Purpose 3) Employment status and religious importance (“Is religion an important part of your daily life?”)	Employment: employed full/part time = 1, out of workforce/unemployed = 0; Religious Importance: yes = 1, no = 0; if either of employment or religious importance = 1, then Purpose 3 = 1
Character	(Character 1) Have you done any of the following in the past month?… Volunteered your time to an organization	Yes = 1 and No = 0
Character	(Character 2) Have you done any of the following in the past month?… Donated money to a charity	Yes = 1 and No = 0
Character	(Character 3) Have you done any of the following in the past month?… Helped a stranger or someone you didn't know who needed help	Yes = 1 and No = 0
Social	(Social 1) If you were in trouble, do you have relatives or friends you can count on to help you whenever you need them, or not?	Yes = 1 and No = 0
Social	(Social 2) Are you satisfied or dissatisfied with the city or area where you live?	Satisfied = 1 and Dissatisfied = 0
Social	(Social 3) Were you treated with respect all day yesterday?	Yes = 1 and No = 0

*Respondents were classified into six categories of employment status (employed full time for an employer, employed full time for self, employed part time and do not want to work full time, employed part time and want to work full time, out of workforce, unemployed) by Gallup based on answers to a series of questions about employment*.

The composite scores for flourishing were calculated as follows. First, binary items were coded such that positive responses (i.e., responses indicating higher levels of wellbeing) were assigned a value of 1, and negative responses were set to 0. Second, we rescaled the two items with responses ranging from 0 to 10 to range from 0 to 1. Third, we took an average of the three items for each domain to obtain domain-specific scores. Fourth, we took an average of the domain-specific scores to obtain a composite index of flourishing. Lastly, we rescaled the composite index by multiplying values by 10. Final scores on the composite flourishing index range from 0 to 10, with greater values indicating higher flourishing levels. Our goal was not to establish flourishing index scores that can be used in future studies but rather to take advantage of the available items in the GWP to roughly quantify multidimensional wellbeing.

We also compared trends for the index of flourishing to those found for Cantril's ladder, a widely used single-item measure of current life satisfaction (12). Cantril's ladder asks respondents to evaluate their current life on a ladder scale ranging from 0 (worst possible life) to 10 (best possible life). Current life satisfaction is one of the three items that formed the happiness domain in our composite flourishing index.

### Statistical Analysis

To assess trends in the distribution of wellbeing, we calculated national average and within-country inequality in flourishing and life satisfaction. We measured inequality in wellbeing using the Gini Index, which summarizes the distribution of wellbeing within the population (18). The Gini Index ranges from 0 (no inequality) to 1 (greatest inequality). Although the Gini Index is often used to assess income inequality, it has also been used in the health disparity literature (19). Other measures of health disparity (e.g., standard deviations) could be used; however, we decided to use the Gini Index because it does not depend on the scale and range of the variable being investigated, which will facilitate comparisons with future studies.

We performed the following analyses. First, we pooled data across years and ranked the 162 participating countries based on mean and Gini Index of flourishing. For the top and bottom 25 countries in both rankings, we compared trends in mean and inequality in composite flourishing and Cantril's ladder life satisfaction. We also compared mean scores for each domain of flourishing across the top and bottom 25 countries. Second, we used the most recent GWP wave (i.e., 2019) to plot and evaluate the relationship between country-specific mean and inequality in composite flourishing and life satisfaction. We also calculated Pearson's correlation of mean and inequality for both wellbeing measures. Third, we examined global trajectories of the mean and inequality for Cantril's ladder, composite flourishing, and each domain of flourishing from 2009 to 2019. We calculated country- and year-specific mean and inequality values for each metric and used their averages as global trends. Fourth, we compared trajectories of mean and inequality in composite flourishing with those in life satisfaction from 2009 to 2019 by geographic regions. Countries were grouped into 11 regions: Africa (Sub-Saharan), Middle East and North Africa, Australia-New Zealand, Commonwealth of Independent States, East Asia, South Asia, Southeast Asia, European Union, Europe (Other), Northern America, and Latin America and the Caribbean. Figure 1 shows countries included in each category. Lastly, to further investigate differential trends across the wellbeing metrics, we pooled the data across years and countries to examine the relationship between age and life satisfaction, composite flourishing, and each domain of flourishing.

We also conducted a series of supplemental analyses. First, we assessed the relationship between flourishing and life satisfaction by producing a scatter plot of country-specific mean values for each wellbeing metric and calculated Pearson's correlation. Second, to delineate more nuanced patterns, we examined trends in mean, inequality, and age relationship in the wellbeing measures by geographic regions. We applied survey weights to all responses to ensure the representativeness of the GWP sample. The survey weights were calculated to adjust for oversampling based on geographic regions, differential survey modes (e.g., landline vs. cellphone), and household size and to standardize the distributions of demographic characteristics (i.e., age, gender, and education) to the 15+ population of each country. All analyses were performed using R, version 3.6.0.

## Results

### Country Ranking

Table 2 shows the top and bottom 25 of 162 countries according to mean composite flourishing scores (see Supplementary Table 2 for the rankings of all countries). Generally, countries ranked highly for mean flourishing also tended to have high mean scores on Cantril's ladder (Pearson's correlation = 0.75 and *p* < 0.01 as shown in Supplementary Figure 1). However, there were some differential trends across the wellbeing metrics. For example, the three countries with the highest mean scores on Cantril's ladder (Denmark, Finland, and Switzerland) were not ranked as highly in terms of flourishing (13th, 22nd, and 12th, respectively). New Zealand, Ireland, and Australia were the three highest-ranked countries in terms of flourishing. The countries with the highest Cantril's ladder values tended to score highest in the happiness domain (e.g., Denmark: 0.83). However, they did not perform as well in other domains compared to the countries with the highest flourishing values (e.g., Denmark vs. New Zealand: 0.77 vs. 0.81 for the purpose domain and 0.44 vs. 0.56 for the character domain).

**Table 2:** Mean composite flourishing, Cantril's ladder score, and each domain of flourishing among top and bottom 25 out of 162 countries according to country ranking for composite flourishing.

**Country**	**Flourishing**		**Cantril's ladder**		**Domain of flourishing, mean (SD)**				
	**Mean (SD)**	**Rank**	**Mean (SD)**	**Rank**	**Happiness**	**Health**	**Purpose**	**Character**	**Social**
**Top 25 in flourishing rank**									
New Zealand	7.71 (1.32)	1	7.31 (1.67)	9	0.80 (0.17)	0.75 (0.29)	0.81 (0.23)	0.56 (0.31)	0.93 (0.16)
Ireland	7.67 (1.33)	2	7.07 (1.77)	15	0.77 (0.18)	0.80 (0.27)	0.78 (0.24)	0.55 (0.32)	0.93 (0.15)
Australia	7.59 (1.37)	3	7.29 (1.71)	10	0.78 (0.18)	0.74 (0.30)	0.77 (0.24)	0.56 (0.30)	0.93 (0.16)
Canada	7.59 (1.37)	4	7.40 (1.67)	7	0.80 (0.17)	0.74 (0.30)	0.81 (0.22)	0.54 (0.31)	0.92 (0.17)
Uzbekistan	7.58 (1.31)	5	5.80 (2.14)	59	0.76 (0.18)	0.78 (0.29)	0.79 (0.22)	0.39 (0.33)	0.94 (0.15)
Iceland	7.55 (1.24)	6	7.45 (1.63)	6	0.81 (0.16)	0.69 (0.32)	0.83 (0.21)	0.48 (0.29)	0.93 (0.15)
Norway	7.52 (1.31)	7	7.53 (1.57)	4	0.81 (0.16)	0.73 (0.30)	0.80 (0.24)	0.46 (0.31)	0.95 (0.13)
United States	7.50 (1.49)	8	7.10 (1.93)	14	0.78 (0.19)	0.73 (0.31)	0.79 (0.23)	0.58 (0.31)	0.88 (0.19)
Netherlands	7.50 (1.34)	9	7.45 (1.30)	5	0.80 (0.15)	0.75 (0.31)	0.74 (0.25)	0.52 (0.29)	0.94 (0.15)
Indonesia	7.50 (1.33)	10	5.25 (1.92)	89	0.70 (0.17)	0.81 (0.25)	0.79 (0.22)	0.50 (0.35)	0.87 (0.21)
Qatar	7.49 (1.23)	11	6.55 (2.03)	27	0.74 (0.20)	0.77 (0.27)	0.83 (0.19)	0.48 (0.29)	0.91 (0.17)
Switzerland	7.46 (1.31)	12	7.55 (1.57)	3	0.78 (0.18)	0.76 (0.29)	0.80 (0.23)	0.44 (0.31)	0.94 (0.14)
Denmark	7.45 (1.26)	13	7.68 (1.58)	1	0.83 (0.15)	0.73 (0.30)	0.77 (0.24)	0.44 (0.29)	0.95 (0.13)
Thailand	7.39 (1.26)	14	6.10 (1.96)	45	0.73 (0.19)	0.77 (0.28)	0.85 (0.20)	0.42 (0.30)	0.87 (0.20)
United Kingdom	7.38 (1.45)	15	6.89 (1.82)	18	0.76 (0.19)	0.76 (0.29)	0.73 (0.26)	0.53 (0.31)	0.91 (0.18)
Austria	7.38 (1.39)	16	7.23 (1.71)	11	0.76 (0.19)	0.78 (0.28)	0.77 (0.24)	0.45 (0.31)	0.93 (0.17)
United Arab Emirates	7.36 (1.30)	17	6.85 (2.01)	20	0.75 (0.20)	0.80 (0.26)	0.82 (0.21)	0.45 (0.32)	0.91 (0.17)
Costa Rica	7.34 (1.33)	18	7.18 (2.16)	13	0.79 (0.19)	0.75 (0.30)	0.85 (0.21)	0.38 (0.32)	0.90 (0.18)
Panama	7.33 (1.34)	19	6.68 (2.46)	24	0.76 (0.20)	0.79 (0.28)	0.85 (0.21)	0.35 (0.35)	0.90 (0.19)
Trinidad and Tobago	7.31 (1.44)	20	6.28 (2.25)	38	0.76 (0.20)	0.73 (0.30)	0.80 (0.22)	0.47 (0.34)	0.86 (0.22)
Sweden	7.30 (1.29)	21	7.36 (1.63)	8	0.80 (0.17)	0.74 (0.30)	0.76 (0.25)	0.39 (0.29)	0.94 (0.15)
Finland	7.28 (1.36)	22	7.56 (1.52)	2	0.77 (0.19)	0.74 (0.30)	0.78 (0.24)	0.40 (0.31)	0.94 (0.15)
Paraguay	7.24 (1.30)	23	5.57 (2.15)	71	0.72 (0.19)	0.79 (0.28)	0.83 (0.22)	0.33 (0.35)	0.92 (0.17)
Puerto Rico	7.24 (1.63)	24	6.82 (2.68)	21	0.76 (0.23)	0.68 (0.32)	0.79 (0.25)	0.40 (0.33)	0.90 (0.18)
Guatemala	7.23 (1.40)	25	6.25 (2.63)	41	0.72 (0.22)	0.75 (0.29)	0.85 (0.21)	0.40 (0.35)	0.88 (0.20)
**Bottom 25 in flourishing rank**									
Gabon	5.80 (1.57)	138	4.43 (2.17)	133	0.55 (0.22)	0.64 (0.32)	0.72 (0.25)	0.27 (0.26)	0.69 (0.28)
Congo Kinshasa	5.79 (1.53)	139	4.35 (1.76)	140	0.58 (0.2)	0.70 (0.31)	0.68 (0.25)	0.21 (0.28)	0.70 (0.27)
Greece	5.76 (1.52)	140	5.53 (2.27)	74	0.60 (0.25)	0.71 (0.30)	0.57 (0.26)	0.17 (0.24)	0.84 (0.24)
Syria	5.74 (1.09)	141	4.10 (2.47)	148	0.48 (0.23)	0.72 (0.26)	0.62 (0.25)	0.38 (0.27)	0.67 (0.29)
Montenegro	5.73 (1.78)	142	5.28 (2.21)	85	0.59 (0.25)	0.67 (0.32)	0.60 (0.29)	0.21 (0.27)	0.79 (0.26)
Iraq	5.73 (1.61)	143	4.71 (2.25)	119	0.52 (0.24)	0.55 (0.33)	0.62 (0.26)	0.33 (0.29)	0.75 (0.27)
Madagascar	5.71 (1.44)	144	3.98 (1.80)	158	0.56 (0.20)	0.65 (0.30)	0.66 (0.25)	0.21 (0.30)	0.77 (0.25)
Palestine	5.71 (1.66)	145	4.62 (2.29)	122	0.55 (0.25)	0.63 (0.34)	0.65 (0.25)	0.20 (0.27)	0.80 (0.25)
Albania	5.70 (1.74)	146	4.99 (2.36)	100	0.60 (0.25)	0.64 (0.34)	0.61 (0.27)	0.23 (0.28)	0.75 (0.27)
Bosnia Herzegovina	5.68 (1.80)	147	5.15 (2.22)	96	0.56 (0.25)	0.64 (0.34)	0.59 (0.28)	0.25 (0.28)	0.76 (0.27)
Egypt	5.67 (1.58)	148	4.45 (2.20)	130	0.52 (0.24)	0.66 (0.33)	0.63 (0.23)	0.24 (0.27)	0.80 (0.24)
Serbia	5.65 (1.82)	149	5.17 (2.25)	94	0.56 (0.26)	0.65 (0.34)	0.60 (0.29)	0.18 (0.26)	0.79 (0.26)
Tunisia	5.63 (1.65)	150	4.78 (2.04)	114	0.56 (0.24)	0.66 (0.32)	0.65 (0.25)	0.25 (0.26)	0.75 (0.28)
Georgia	5.61 (1.80)	151	4.23 (2.01)	146	0.53 (0.25)	0.60 (0.37)	0.61 (0.25)	0.21 (0.26)	0.74 (0.26)
Nagorno Karabakh	5.55 (1.69)	152	4.86 (1.70)	110	0.54 (0.23)	0.58 (0.34)	0.68 (0.24)	0.23 (0.25)	0.74 (0.26)
Haiti	5.54 (1.73)	153	3.96 (2.16)	159	0.48 (0.22)	0.63 (0.33)	0.63 (0.27)	0.42 (0.34)	0.60 (0.31)
Benin	5.53 (1.58)	154	4.00 (2.37)	155	0.55 (0.22)	0.60 (0.32)	0.74 (0.24)	0.24 (0.29)	0.62 (0·30)
Chad	5.52 (1.60)	155	4.04 (2.12)	153	0.53 (0.21)	0.61 (0.33)	0.67 (0.24)	0.26 (0.30)	0.68 (0.30)
Yemen	5.50 (1.66)	156	4.00 (2.31)	156	0.51 (0.26)	0.66 (0.33)	0.64 (0.23)	0.19 (0.25)	0.76 (0.27)
Afghanistan	5.45 (1.85)	157	3.66 (1.85)	164	0.47 (0.23)	0.69 (0.32)	0.62 (0.25)	0.31 (0.30)	0.63 (0.30)
Armenia	5.40 (1.77)	158	4.52 (2.16)	125	0.51 (0.26)	0.57 (0.35)	0.61 (0.27)	0.23 (0.25)	0.75 (0.26)
Central African Republic	5.33 (1.55)	159	3.52 (2.10)	167	0.50 (0.22)	0.57 (0.34)	0.71 (0.23)	0.30 (0.31)	0.62 (0.28)
Togo	5.31 (1.62)	160	3.56 (2.31)	165	0.51 (0.22)	0.58 (0.32)	0.72 (0.25)	0.24 (0.29)	0.57 (0.31)
Burundi	5.26 (1.63)	161	3.55 (2.04)	166	0.52 (0.22)	0.69 (0.32)	0.66 (0.26)	0.14 (0.25)	0.58 (0.31)
South Sudan	5.22 (1.80)	162	3.40 (3.08)	168	0.45 (0.26)	0.51 (0.33)	0.66 (0.26)	0.37 (0.34)	0.60 (0.30)

*Data were pooled across all waves (2009–2019). Scores for composite flourishing and Cantril's Ladder ranged from 0 to 10. Scores for each domain of flourishing ranged from 0 to 1. Higher scores indicate better wellbeing*.

Some countries had an even larger discrepancy between flourishing and Cantril's ladder (e.g., Indonesia: 10th on mean flourishing vs. 89th on mean Cantril's ladder; Greece: 140th on mean flourishing vs. 74th on mean Cantril's ladder), often pertaining to differences in the purpose and character domains. Moreover, countries with similar levels of mean composite flourishing performed differently across the domains of flourishing.

**Figure 2:**
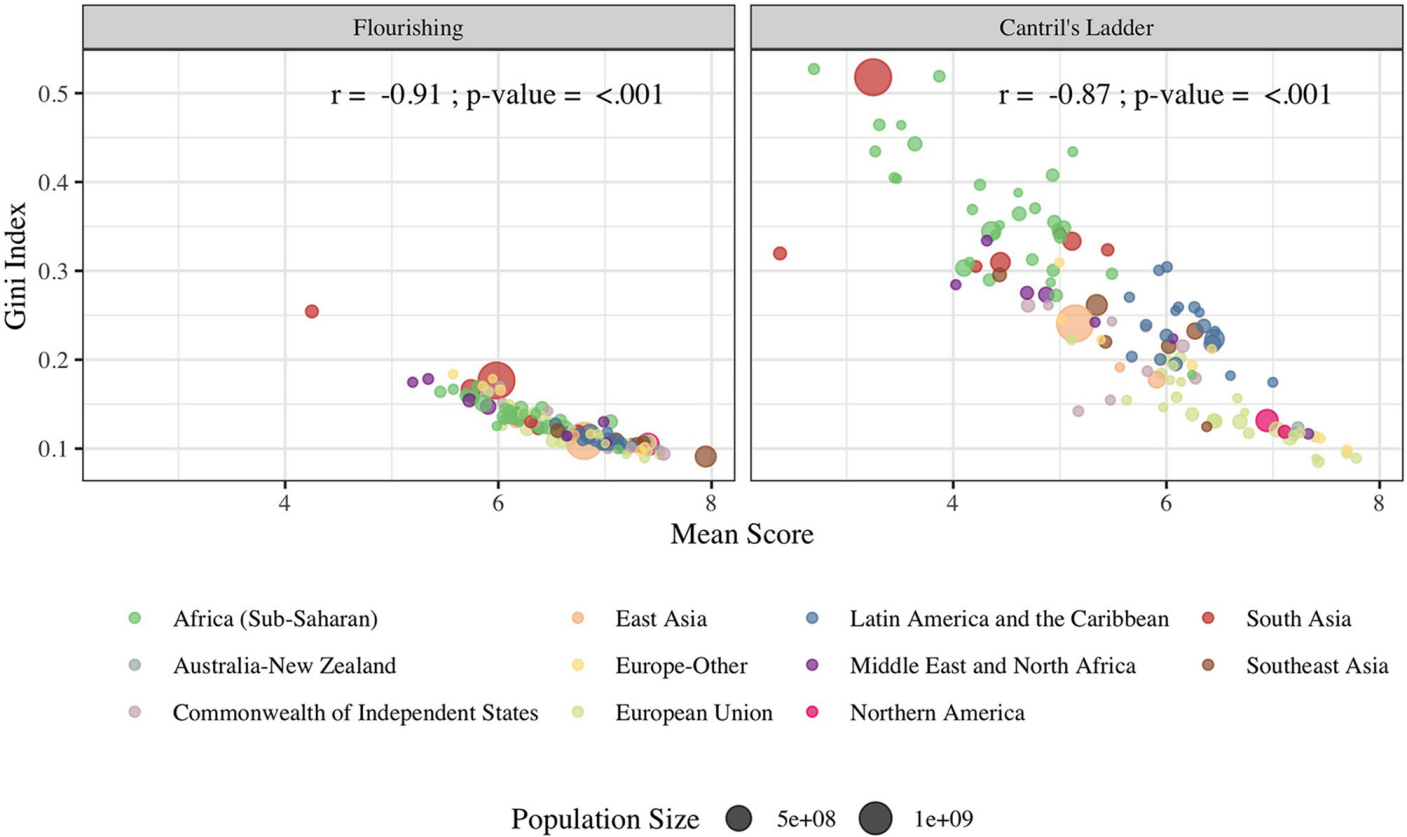
Correlations Between Country-specific Mean Scores and Gini Indices in 2019. Pearson's correlation coefficients were calculated. Points were colored by regions. Size of each point represents the country's population size.

Table 3 shows the top and bottom 25 countries according to inequalities (Gini Index) in composite flourishing (see Supplementary Table 3 for the rankings of all countries). The inequality ranking was largely comparable with the ranking for mean flourishing. As shown in Figure 2, countries with higher mean scores tended to have lower inequalities in wellbeing (Pearson's correlations between country mean scores and Gini Indexes: −0.91, *p* = 0.01 for flourishing; −0.87, *p* = 0.01 for Cantril's ladder). However, some countries ranked highly in mean flourishing (e.g., Australia, 3rd; the United States, 8th) ranked lower on the Gini Index (15th and 29th, respectively).

**Table 3:** Gini index of composite flourishing, Cantril's ladder score, and each domain of flourishing among top and bottom 25 out of 162 countries according to country ranking for composite flourishing.

**Country**	**Flourishing**		**Cantril's ladder**		**Domain of flourishing (Gini index)**				
	**Gini index**	**Rank**	**Gini index**	**Rank**	**Happiness**	**Health**	**Purpose**	**Character**	**Social**
**Top 25 in flourishing rank**									
Qatar	0.09	1	0.17	32	0.15	0.18	0.12	0.32	0.07
Iceland	0.09	2	0.12	8	0.10	0.24	0.12	0.33	0.06
Denmark	0.09	3	0.11	3	0.09	0.22	0.16	0.34	0.05
New Zealand	0.09	4	0.12	11	0.11	0.20	0.15	0.30	0.06
Thailand	0.10	5	0.17	37	0.14	0.19	0.12	0.38	0.11
Ireland	0.10	6	0.14	17	0.12	0.17	0.15	0.31	0.06
Norway	0.10	7	0.11	4	0.10	0.21	0.15	0.36	0.04
United Arab Emirates	0.10	8	0.16	25	0.14	0.16	0.13	0.37	0.07
Sweden	0.10	9	0.12	9	0.11	0.21	0.17	0.38	0.06
Uzbekistan	0.10	10	0.20	65	0.13	0.20	0.14	0.47	0.06
Switzerland	0.10	11	0.11	5	0.12	0.19	0.14	0.37	0.05
Netherlands	0.10	12	0.09	1	0.09	0.20	0.17	0.30	0.06
Indonesia	0.10	13	0.20	60	0.13	0.15	0.14	0.38	0.11
Canada	0.10	14	0.12	10	0.11	0.21	0.14	0.31	0.07
Australia	0.10	15	0.12	12	0.12	0.21	0.16	0.29	0.06
Luxembourg	0.10	16	0.11	6	0.13	0.19	0.17	0.42	0.06
Paraguay	0.10	17	0.22	82	0.15	0.19	0.13	0.56	0.08
Bhutan	0.10	18	0.12	13	0.12	0.17	0.13	0.37	0.14
Costa Rica	0.10	19	0.17	30	0.13	0.21	0.12	0.47	0.09
Panama	0.10	20	0.20	73	0.14	0.18	0.12	0.54	0.09
Austria	0.10	21	0.13	15	0.14	0.18	0.16	0.37	0.07
Malta	0.10	22	0.17	31	0.19	0.20	0.13	0.34	0.08
Finland	0.10	23	0.10	2	0.13	0.21	0.16	0.43	0.05
Philippines	0.11	24	0.26	127	0.18	0.22	0.10	0.47	0.10
Turkmenistan	0.11	25	0.17	36	0.16	0.20	0.18	0.40	0.08
**Bottom 25 in flourishing rank**									
Chad	0.16	138	0.28	139	0.22	0.28	0.19	0.56	0.23
Lebanon	0.16	139	0.25	113	0.28	0.23	0.21	0.52	0.15
Lithuania	0.16	140	0.19	57	0.22	0.25	0.25	0.65	0.13
Benin	0.16	141	0.31	153	0.22	0.29	0.17	0.60	0.26
Algeria	0.16	142	0.19	51	0.21	0.23	0.21	0.59	0.17
Central African Republic	0.16	143	0.32	155	0.25	0.32	0.17	0.54	0.24
Ukraine	0.16	144	0.24	101	0.24	0.30	0.25	0.63	0.13
Tunisia	0.16	145	0.23	95	0.24	0.26	0.21	0.52	0.19
Palestine	0.17	146	0.27	131	0.26	0.30	0.21	0.62	0.15
North Macedonia	0.17	147	0.25	111	0.25	0.26	0.21	0.61	0.16
Croatia	0.17	148	0.19	52	0.23	0.25	0.22	0.66	0.16
Nagorno Karabakh	0.17	149	0.18	44	0.24	0.32	0.18	0.53	0.18
Togo	0.17	150	0.34	161	0.24	0.29	0.18	0.60	0.29
Yemen	0.17	151	0.32	157	0.29	0.27	0.19	0.60	0.18
Bulgaria	0.17	152	0.26	119	0.28	0.27	0.27	0.65	0.12
Montenegro	0.17	153	0.23	94	0.24	0.25	0.25	0.62	0.16
Albania	0.17	154	0·26	124	0.23	0.30	0.23	0.61	0.18
Burundi	0.17	155	0.31	151	0.25	0.24	0.21	0.75	0.29
Haiti	0.18	156	0.30	148	0.27	0.29	0.23	0.44	0.28
Bosnia Herzegovina	0.18	157	0.24	99	0.25	0.28	0.25	0.58	0.18
Serbia	0.18	158	0.25	109	0.27	0.28	0.26	0.68	0.17
Georgia	0.19	159	0.27	130	0.28	0.35	0.22	0.62	0.18
South Sudan	0.19	160	0.48	168	0.33	0.35	0.21	0.46	0.27
Afghanistan	0.19	161	0.28	138	0.27	0.24	0.21	0.51	0.26
Armenia	0.19	162	0.27	133	0.30	0.36	0.24	0.58	0.18

*Data were pooled across all waves (2009–2019). A higher rank for the Gini Index indicates less inequality in wellbeing*.

### Temporal Trends in Wellbeing

**Figure 3:**
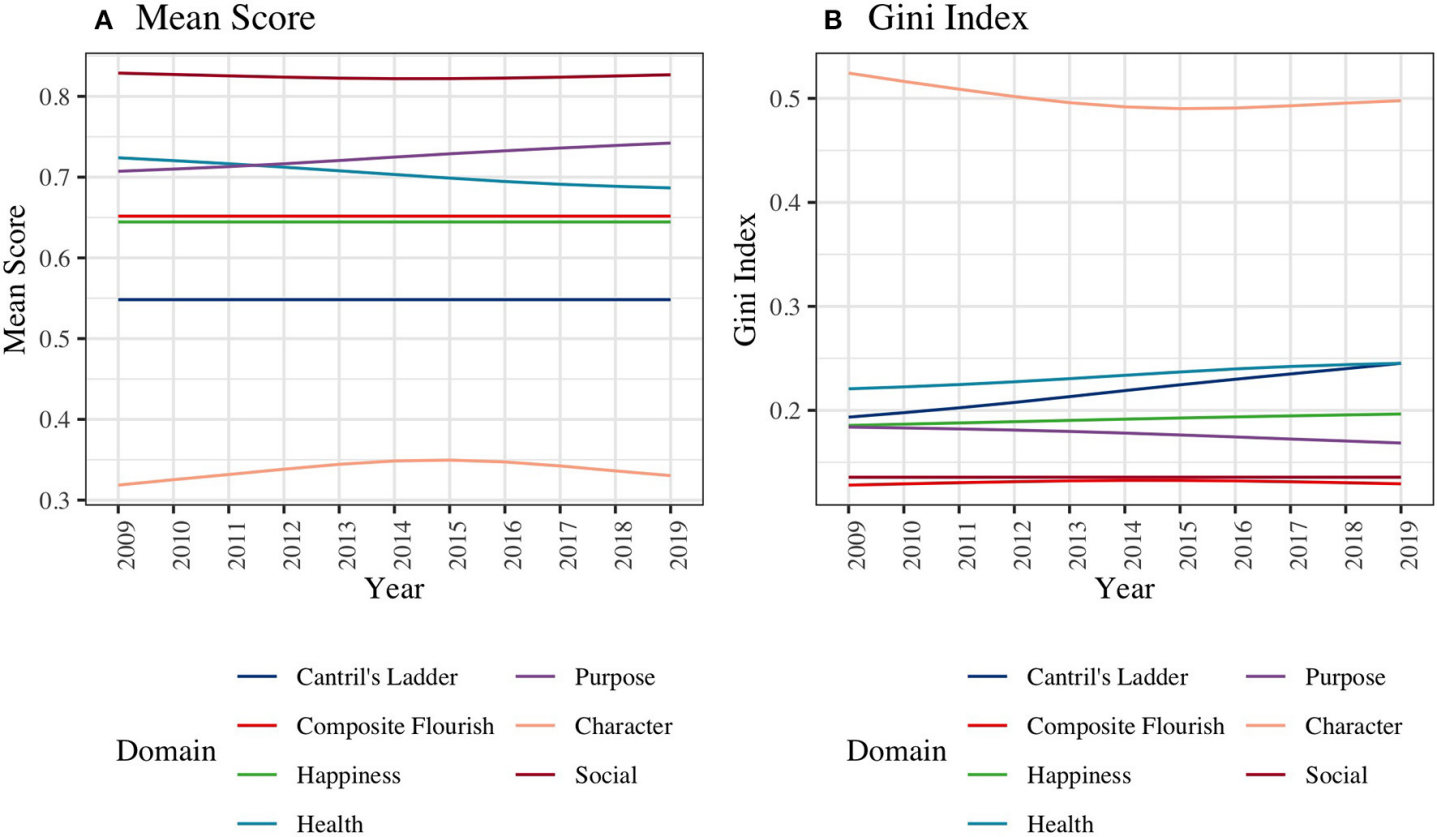
Global Trend of **(A)** Mean and **(B)** Inequality for the Composite Flourishing and Each Domain of Flourishing from 2009 to 2019. To facilitate comparison with the domain-specific scores, the mean composite flourishing scores and Cantril's Ladder scale scores were re-scaled to range from 0 to 1. We computed mean scores and Gini indices of all the wellbeing measures for each country and year and then plotted smoothed curves for global trajectories.

Figure 3 shows the global trajectories of mean scores and Gini Indexes for composite flourishing and Cantril's ladder. Mean scores for composite flourishing and Cantril's ladder were both stable over time. Regarding inequality of wellbeing, the Gini Indexes for Cantril's ladder showed a monotonic increase (0.20 in 2009 to 0.25 in 2019) but remained somewhat more constant for composite flourishing. For domains of flourishing, we found increasing population average scores and decreasing inequality in the purpose domain (Average: 0.71 in 2009 to 0.74 in 2019; Gini Index: 0.18 in 2009 to 0.17 in 2019). In contrast, we found decreasing population average scores and increasing inequality in the health domain (Average: 0.72 in 2009 to 0.69 in 2019; Gini Index: 0.23–0.24).

**Figure 4:**
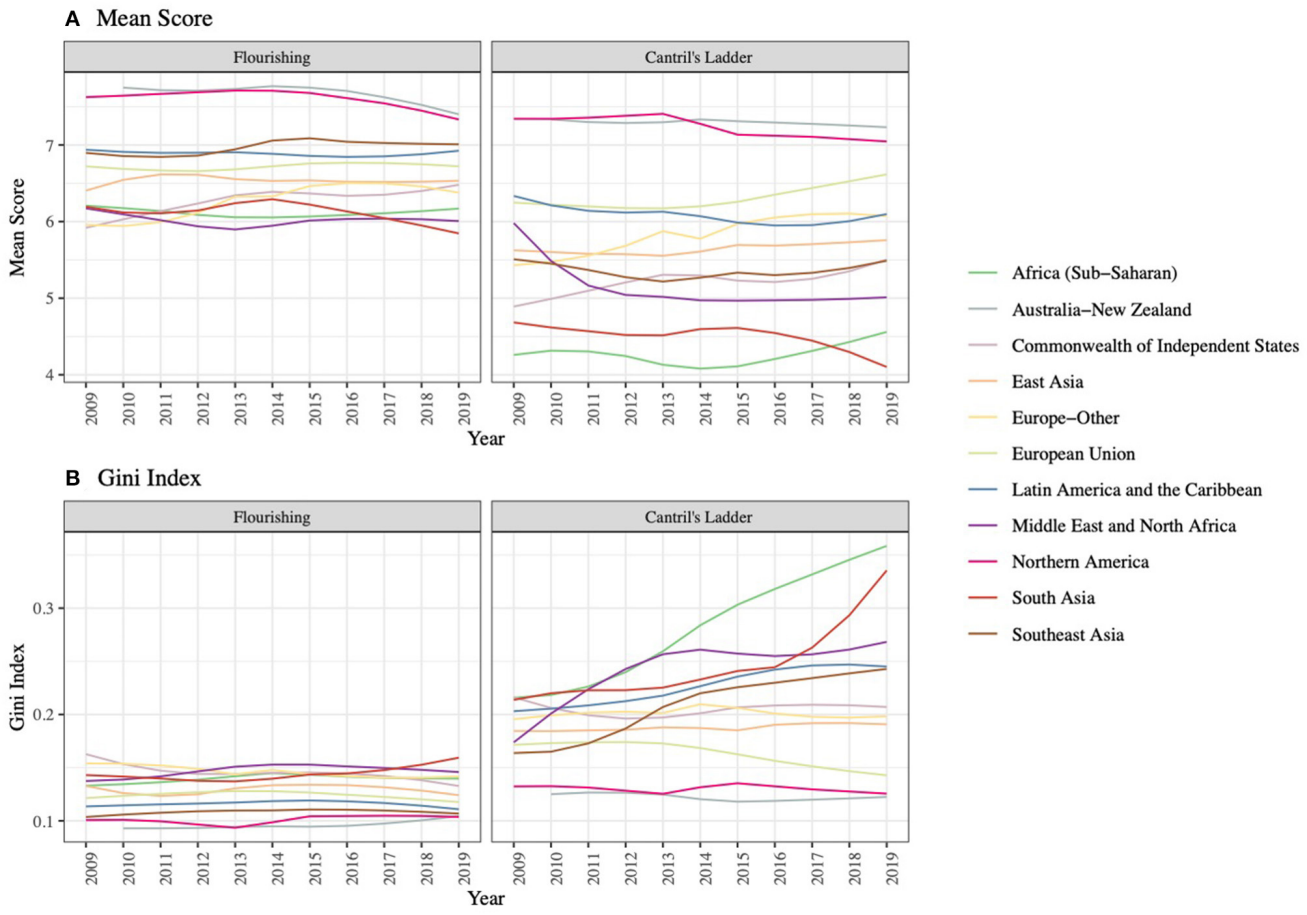
Trajectories of **(A)** Mean Score and **(B)** Gini Index for Flourishing and Cantril's Ladder from 2009 to 2019 by Regions. We computed mean scores and Gini indices of the composite flourishing and Cantril's ladder for each country and year and then plotted smoothed curves for trajectories in each region.

Figure 4 shows region-specific trajectories of mean scores and Gini Indexes of composite flourishing and Cantril's ladder. For both flourishing and Cantril's ladder, mean scores were relatively stable over time across regions and highest in Australia-New Zealand and Northern America. The Gini Index for flourishing was relatively stable and lowest in Australia-New Zealand and Northern America. However, when assessing Cantril's ladder, there was a monotonic increase in the Gini Indexes in Sub-Saharan Africa (0.21 in 2009 to 0.36 in 2019), Middle East and North Africa (0.19 in 2009 to 0.25 in 2019), South Asia (0.22 in 2009 to 0.35 in 2019), and Latin America and the Caribbean (0.20 in 2009 to 0.24 in 2019). In other regions, the Gini Indexes for Cantril's ladder remained constant or even decreased. Region-specific trajectories for each domain of flourishing are presented in Supplementary Figures 2, 3. Overall, the temporal trends were relatively similar across regions but differed across domains.

### Age and Wellbeing

**Figure 5:**
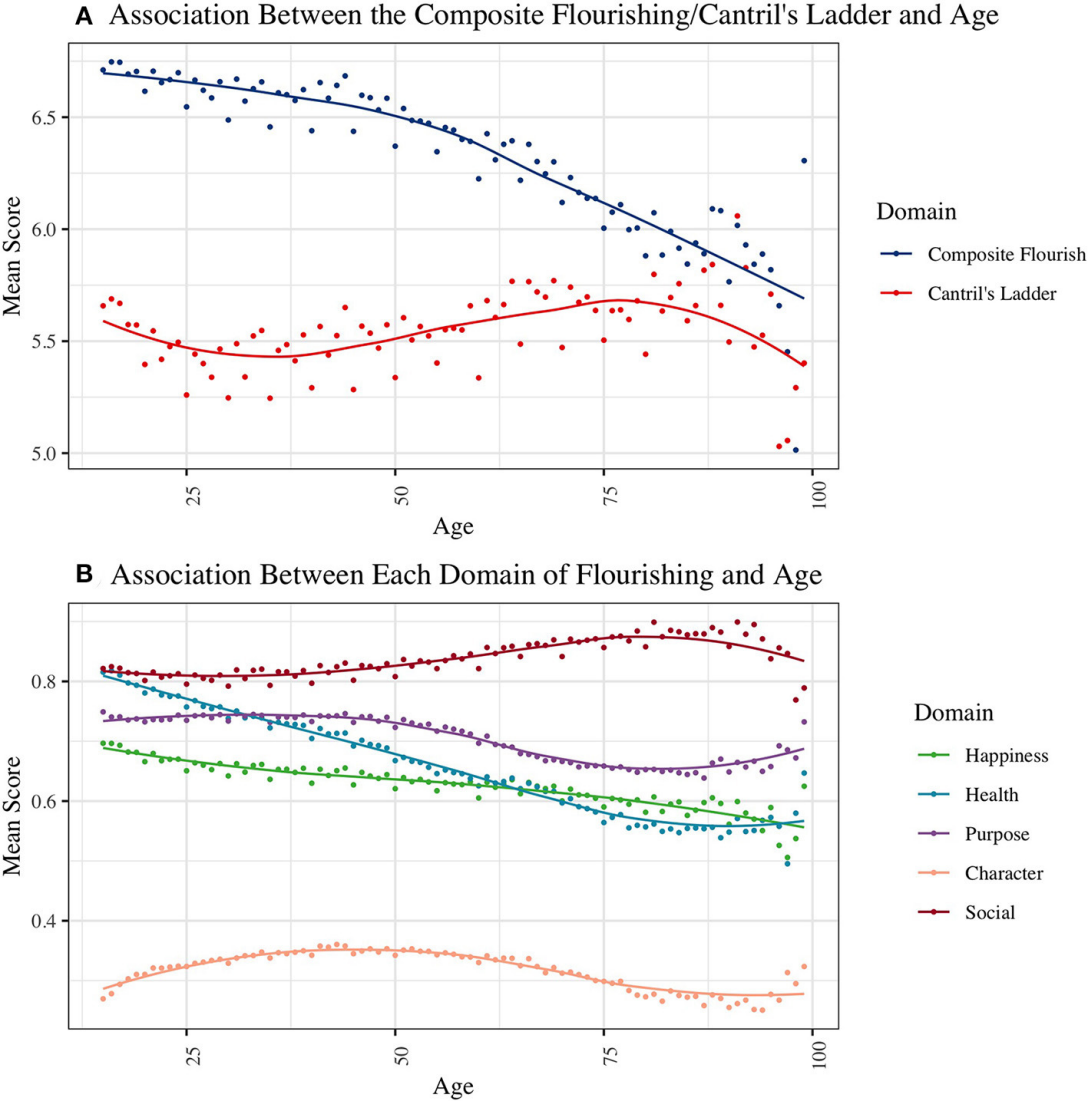
Associations of **(A)** Composite Flourishing/Cantril's Ladder and **(B)** Each Domain of Flourishing with Age. Data was pooled across countries and years.

Figure 5 shows the relationship between age and mean scores for each wellbeing metric. We found that the mean for Cantril's ladder decreased with age until early adulthood, increased with age after early adulthood, and declined again in very late life (after age 80). Conversely, the mean composite flourishing score monotonically decreased across the life-course. Region-specific age trends are shown in Supplementary Figure 4. For both composite flourishing and Cantril's ladder, relationships with age varied to some extent across regions. Regarding specific domains of flourishing, mean scores for the health domain and happiness domain (which included life satisfaction and affect) decreased with age. In contrast, scores for the social wellbeing domain increased with age. Mean values of the purpose and character domains peaked in middle age (around 40–50s) but declined in later life.

## Discussion

This study investigated global trends in wellbeing from 2009 to 2019. Prior work in the field has primarily focused on population averages in wellbeing based on a unidimensional metric, such as Cantril's ladder as an index of life satisfaction. This study adds to the existing evidence by examining multidimensional wellbeing (i.e., flourishing) and assessing both population averages and inequalities in wellbeing. To do so, we leveraged data from the GWP, a nationally representative sample of 1.2 million individuals from 162 countries with a wide range of wellbeing measures. Our study has three main findings:

1. We observed a positive correlation between the mean composite flourishing scores and mean Cantril's ladder scores and a negative correlation between mean scores and the degree of inequality assessed by the Gini Index for both flourishing and Cantril's ladder.
2. Despite the correlation between the two metrics of wellbeing, there were distinct patterns in the multidimensional flourishing concerning time, geography, and age that were not evident when wellbeing was assessed using Cantril's ladder as a unidimensional measure of wellbeing.
3. Despite the correlation between the mean scores and Gini Indexes, we identified non-trivial trends in inequalities of wellbeing that were not detected when analyzing average wellbeing.

### Flourishing vs. Cantril's Ladder

The comparison of trends in the multidimensional flourishing with those in the Cantril's ladder scores gave us at least two major insights. First, assessing wellbeing using Cantril's ladder did not appear to sufficiently capture the multidimensional nature of flourishing. The positive correlation between composite flourishing and Cantril's ladder suggests value in using Cantril's ladder as a simple approach to capture wellbeing trends. However, there were notable differences between trends in composite flourishing and Cantril's ladder for many countries. Some countries had higher mean levels of Cantril's ladder relative to composite flourishing (e.g., Scandinavian countries and Greece). Many of those countries scored highly on the happiness domain, for which current life satisfaction constituted one item of the index but did not score as highly in other flourishing domains (e.g., purpose and character). We speculate that this trend may reflect the relatively lower levels of individualism in those countries (20). Similarly, countries with higher levels of composite flourishing levels relative to Cantril's ladder (e.g., Indonesia) scored lower in the happiness domain but higher in other domains (e.g., health, purpose, and character). The relationships with age were remarkably different between Cantril's ladder and composite flourishing and across the flourishing domains, suggesting wellbeing is not a unidimensional construct. Our finding is consistent with the prior work by Huppert and So (21) that analyzed a representative sample of 43,000 Europeans and examined the relationship between their flourishing measure and life evaluation. They found a relatively low correlation between flourishing and life evaluation, although their flourishing measure differs from ours in that their measure more extensively covered aspects of psychological wellbeing (e.g., optimism and vitality) but omitted some domains that we examined (health and character). Taken together, the findings imply that a unidimensional Cantril's ladder may adequately assess one dimension of flourishing (i.e., happiness) but fails to identify differential trends in other essential domains of flourishing.

Second, even the composite flourishing appeared inadequate and needed to be complemented with independent evaluations of each domain to obtain a full picture of flourishing. Countries with similar mean composite flourishing levels had different domain-specific wellbeing levels, suggesting they require different interventions to promote flourishing. Further, although the global mean level of composite flourishing has remained constant over time, wellbeing increased in the purpose domain and decreased in the health domain during the same period. Collectively, these findings suggest that the specific domains of flourishing to be prioritized for the promotion of complete wellbeing did change over time. For interventions to be more targeted at the changing needs of wellbeing globally, it is therefore important to assess flourishing as a composite as well as with each of the domains.

Previous research focused on Cantril's ladder has resulted in insufficient attention to how the interrelationships between purpose, character, and health shape global wellbeing. We found that scores on these three domains decline with age, suggesting that interventions might be particularly helpful for older adults in rapidly aging societies (22). Accumulating evidence suggests that some of the items we used to measure purpose and character have important effects on population health. For example, self-reported purpose is associated with lower risk of mortality and increased likelihood of engaging in preventive behaviors (23–26). Similarly, volunteering, one of our character items, has been found to predict lower risk of mortality, higher physical activity, and a range of wellbeing outcomes, including greater purpose (27). More research is warranted to better understand how these three domains impact global wellbeing.

### Population Average vs. Inequality

Although population average scores and Gini index were negatively correlated, some countries (e.g., Australia and the United States) with high population average flourishing had relatively large inequality. We also identified rapidly rising inequalities in life satisfaction in Africa, South Asia, Latin America, and the Caribbean, which might be explained partly by the unequal distributions of life satisfaction drivers (e.g., income) in these countries. Our findings indicate that wellbeing inequalities can arise even when the population average wellbeing appears high or stable over time. Notably, the analysis of average population wellbeing did not provide these insights.

To inform policies for reducing wellbeing inequalities, it is important to identify the drivers of such inequalities and how they differ across populations. The wellbeing inequalities observed in this study might reflect increasing social inequalities within some countries. While socioeconomic conditions have been documented to affect multiple domains of wellbeing in many studies, the relationship might be heterogeneous across populations. For instance, studies based on European populations demonstrated that socioeconomic factors (e.g., education) were associated with mental illness but did not predict mental wellbeing (28, 29). Because our measure of inequality (Gini Index) summarized overall inequalities within populations, future studies are warranted to assess inequalities based on specific social groups (e.g., does flourishing differ across income groups?).

While it is informative to study determinants of flourishing that can be altered *via* interventions targeting individuals, future studies should also examine more social and structural determinants of population wellbeing (e.g., policies and neighborhood environment). Interventions that aim to shift the distribution of flourishing within the whole population may be a more effective public health strategy than intervening among “high-risk” people at the individual level, although the two approaches are not mutually exclusive (30). Such population-level approaches may also narrow existing inequalities in wellbeing (31), as indicated by the observed negative correlations between population averages and inequalities in this study.

### Future Directions of Wellbeing Research

Our study has three implications for future global research on wellbeing. First, a multidimensional approach to assessing wellbeing is likely to better inform policies aimed at enhancing human wellbeing more holistically. We showed that unidimensional life satisfaction wellbeing assessment -a common approach in the literature- may overlook important trends in other critical flourishing domains. Because trends can vary substantially across domains of flourishing, future efforts dedicated to tracking wellbeing are encouraged to assess composite flourishing as well as domain-specific wellbeing.

In studying determinants or impacts of policies on population wellbeing, assessing domain-specific wellbeing using multiple items is helpful because a specific exposure/treatment can promote wellbeing in one domain and deteriorate wellbeing in other domains. For instance, evidence suggests that taking care of children as an expression of the self may improve a sense of meaning but can decrease happiness (32). We also need a better understanding of the determinants of the domains of wellbeing for which empirical research is scarce (e.g., purpose or character). Research in this area could provide some explanations about the distinct trends in Cantril's ladder and flourishing that we observed in this study. One useful approach for such a study is to conduct an outcome-wide analysis, which examines the effects of a single factor on a wide range of outcomes simultaneously (33). This approach has several methodological advantages (e.g., preventing p-hacking and publication bias) and provides a holistic view of the relationships between the exposure/treatment and subsequent wellbeing.

Second, more comprehensive measurements for the flourishing domains and inclusion of those items during data collection are warranted. Most datasets used in public health and social science research do not contain a full set of items to assess flourishing. Thus, a feasible approach has been to use available items as a proxy to characterize the flourishing domain of interest, as we did in this study. Given the theoretical advantage of using multidimensional measures to more comprehensively capture relevant domains of human wellbeing, the quality of future research in this area will rest on the development, evaluation, and refinement of psychometrically sound measures of multidimensional wellbeing that are validated for use within and across different cultural contexts.

Lastly, future research should go beyond assessing only average population wellbeing and also pay attention to inequalities of wellbeing within populations. Tracking population averages may not be particularly informative for a more equitable promotion of wellbeing because, as we demonstrated, existing and widening inequalities may be masked in such an analysis. Although we used Gini Index as a simple measure of total inequality in a population, future studies are encouraged to conduct more comprehensive assessment that considers (a) inequality across specific social groups (e.g., inequality in wellbeing across levels of income) and (b) the scale of inequality measure (i.e., relative vs. absolute inequality) (19).

### Study Limitations

Three limitations should be noted. First, our flourishing measures were admittedly crude. Because of the limited data availability of GWP items, some items were, as noted above, a proxy of the flourishing domain of interest at best. For example, we assessed the character domain by measuring whether subjects engaged in volunteering, donating money, and helping others in the past month. These arguably do not constitute character itself but rather are its consequences. Although we may expect these measures to reflect one's character well, other facilitators of and barriers to these behaviors may exist, such as social norms, transportation, physical health, and financial conditions. In assessing the purpose domain, we used one's employment status and religious importance, but some individuals may not derive purpose from these conditions. The items for the purpose domain and the character domain showed weak within-domain correlations. Furthermore, other important theorizing around multidimensional wellbeing exists [e.g., works by (3, 34–39)]. Although we calculated continuous composite scores for each domain, the domain-specific scores were based exclusively on three individual binary items (except for the happiness domain), which do not capture the nuanced levels of wellbeing. Moreover, the scores were calculated as simple averages of the individual items, thereby assigning equal weight to each item. However, this study's purpose was not to establish a comprehensive measure of flourishing but rather to use the GWP data as best as possible. We did not intend to be prescriptive of what constitutes a “good” life by choosing the specific items (e.g., working and religion) either. We aimed to compare trends in wellbeing assessed multidimensionally with those from a unidimensional wellbeing measure, hoping to create a foundation that facilitates future research with more rigorous multidimensional wellbeing measures. Our finding also warrants future studies using other existing data sources that examine wellbeing globally using different items (e.g., World Values Survey). Second, differences in culture and language may alter people's perceptions of the world (40–42). International comparisons involving metrics such as life satisfaction rely on equivalence among translations. Despite scholars' best efforts, there are often at least subtle differences between translated terms. Moreover, cultural variation affects how people respond to surveys; participants in individualistic contexts tend to emphasize their positive feelings, whereas respondents in more collectivist locales may be more likely to downplay these in a self-effacing manner (43). Third, the regional grouping we used to categorize countries may be crude. Other approaches to categorize countries (e.g., eastern vs. western vs. southern Europe for countries in Europe) exist, although any grouping of countries, including ours, can be arbitrary and imperfect.

## Conclusions

In conclusion, the current study demonstrated that the standard approach to monitoring wellbeing that assesses population averages using a unidimensional measure might not fully capture complex and changing patterns in wellbeing. Achieving complete wellbeing (i.e., human flourishing) is the ultimate goal of many international organizations and global initiatives. We propose that future research on wellbeing needs to establish and use more comprehensive multidimensional wellbeing measures and assess population distributions of wellbeing. Such research would inform a more effective and equitable promotion of human flourishing.

## Data Availability Statement

De-identified data that underlie the results reported in this article will be available upon request to and permission by the Gallup Inc. Programming code to replicate the study findings will be available upon request to Koichiro Shiba. Requests to access these datasets should be directed to shiba_k@g.harvard.edu.

## Author Contributions

KS was responsible for conceptualization, data curation, formal analysis, funding acquisition, investigation, methodology, project administration, and writing an original draft. RC, ML, and TV were responsible for methodology, reviewing, and editing the draft. NG, TL, and AL conducted literature review and reviewing and editing of the draft. All authors contributed to the article and approved the submitted version.

## Funding

This study was supported by the Wellbeing for Planet Earth Foundation. TV was funded by the John Templeton Foundation (Grant Number 61075).

## Conflict of Interest

The authors declare that the research was conducted in the absence of any commercial or financial relationships that could be construed as a potential conflict of interest.

## Publisher's Note

All claims expressed in this article are solely those of the authors and do not necessarily represent those of their affiliated organizations, or those of the publisher, the editors and the reviewers. Any product that may be evaluated in this article, or claim that may be made by its manufacturer, is not guaranteed or endorsed by the publisher.
