# Supplementary material for: Global Trends of Mean and Inequality in Multidimensional Wellbeing: Analysis of 1.2 Million Individuals From 162 Countries, 2009–2019
Source: Front Public Health. 2022 Feb 14;10:824960. doi: 10.3389/fpubh.2022.824960 (PMC8882600; doi:10.3389/fpubh.2022.824960)
Supplement: Supplementary file 1 [file Data_Sheet_1.docx]

**eTable 1. Correlations Between Items for the Composite Flourishing Score**

|  | **Happiness 1** | **Happiness 2** | **Happiness 3** | **Health 1** | **Health 2** | **Health 3** | **Purpose 1** | **Purpose 2** | **Purpose 3** | **Character 1** | **Character 3** | **Character 3** | **Social 1** | **Social 2** | **Social 3** |
| --- | --- | --- | --- | --- | --- | --- | --- | --- | --- | --- | --- | --- | --- | --- | --- |
| **Happiness 1** | 1 | ·· | ·· | ·· | ·· | ·· | ·· | ·· | ·· | ·· | ·· | ·· | ·· | ·· | ·· |
| **Happiness 2** | 0·61 | 1 | ·· | ·· | ·· | ·· | ·· | ·· | ·· | ·· | ·· | ·· | ·· | ·· | ·· |
| **Happiness 3** | 0·18 | 0·17 | 1 | ·· | ·· | ·· | ·· | ·· | ·· | ·· | ·· | ·· | ·· | ·· | ·· |
| **Health 1** | 0·14 | 0·18 | 0·12 | 1 | ·· | ·· | ·· | ·· | ·· | ·· | ·· | ·· | ·· | ·· | ·· |
| **Health 2** | 0·12 | 0·14 | 0·18 | 0·29 | 1 | ·· | ·· | ·· | ·· | ·· | ·· | ·· | ·· | ·· | ·· |
| **Health 3** | 0·13 | 0·11 | 0·28 | 0·09 | 0·21 | 1 | ·· | ·· | ·· | ·· | ·· | ·· | ·· | ·· | ·· |
| **Purpose 1** | 0·11 | 0·11 | 0·11 | 0·04 | 0·05 | 0·1 | 1 | ·· | ·· | ·· | ·· | ·· | ·· | ·· | ·· |
| **Purpose 2** | 0·14 | 0·15 | 0·26 | 0·09 | 0·08 | 0·13 | 0·06 | 1 | ·· | ·· | ·· | ·· | ·· | ·· | ·· |
| **Purpose 3** | 0·02 | 0·04 | 0·03 | 0·03 | 0 | -0·02 | 0·03 | 0·03 | 1 | ·· | ·· | ·· | ·· | ·· | ·· |
| **Character 1** | 0·05 | 0·05 | 0·05 | 0 | 0 | 0·01 | 0·02 | 0·10 | 0·03 | 1 | ·· | ·· | ·· | ·· | ·· |
| **Character 2** | 0·08 | 0·06 | 0·07 | -0·01 | 0 | 0·02 | 0·04 | 0·09 | 0·04 | 0·24 | 1 | ·· | ·· | ·· | ·· |
| **Character 3** | 0·05 | 0·08 | 0·07 | 0·02 | -0·01 | -0·01 | 0·04 | 0·11 | 0·06 | 0·21 | 0·21 | 1 | ·· | ·· | ·· |
| **Social 1** | 0·17 | 0·17 | 0·12 | 0·10 | 0·09 | 0·10 | 0·08 | 0·09 | 0·02 | 0·02 | 0·04 | 0·04 | 1 | ·· | ·· |
| **Social 2** | 0·14 | 0·10 | 0·13 | 0·03 | 0·07 | 0·13 | 0·18 | 0·07 | 0·03 | 0·01 | 0·04 | 0·01 | 0·09 | 1 | ·· |
| **Social 3** | 0·10 | 0·09 | 0·20 | 0·05 | 0·08 | 0·20 | 0·12 | 0·12 | 0·03 | 0·01 | 0·03 | 0·03 | 0·10 | 0·12 | 1 |

Correlations were calculated within each country and then averaged across countries.

**eTable 2. Mean Composite Flourishing, Cantril’s Ladder Score, and Each Domain of Flourishing Among 162 Countries**

| **Country** | **Flourishing (Mean)** | **Flourishing (Rank)** | **Cantril’s Ladder**  **(Mean)** | **Cantril’s Ladder (Rank)** | **Domain of Flourishing (Mean)** | | | | |
| --- | --- | --- | --- | --- | --- | --- | --- | --- | --- |
|  |  |  |  |  | **Happiness** | **Health** | **Purpose** | **Character** | **Social** |
| New Zealand | 7·71 | 1 | 7·31 | 9 | 0·8 | 0·75 | 0·81 | 0·56 | 0·93 |
| Ireland | 7·67 | 2 | 7·07 | 15 | 0·77 | 0·8 | 0·78 | 0·55 | 0·93 |
| Australia | 7·59 | 3 | 7·29 | 10 | 0·78 | 0·74 | 0·77 | 0·56 | 0·93 |
| Canada | 7·59 | 4 | 7·4 | 7 | 0·8 | 0·74 | 0·81 | 0·54 | 0·92 |
| Uzbekistan | 7·58 | 5 | 5·8 | 59 | 0·76 | 0·78 | 0·79 | 0·39 | 0·94 |
| Iceland | 7·55 | 6 | 7·45 | 6 | 0·81 | 0·69 | 0·83 | 0·48 | 0·93 |
| Norway | 7·52 | 7 | 7·53 | 4 | 0·81 | 0·73 | 0·8 | 0·46 | 0·95 |
| United States | 7·5 | 8 | 7·1 | 14 | 0·78 | 0·73 | 0·79 | 0·58 | 0·88 |
| Netherlands | 7·5 | 9 | 7·45 | 5 | 0·8 | 0·75 | 0·74 | 0·52 | 0·94 |
| Indonesia | 7·5 | 10 | 5·25 | 89 | 0·7 | 0·81 | 0·79 | 0·5 | 0·87 |
| Qatar | 7·49 | 11 | 6·55 | 27 | 0·74 | 0·77 | 0·83 | 0·48 | 0·91 |
| Switzerland | 7·46 | 12 | 7·55 | 3 | 0·78 | 0·76 | 0·8 | 0·44 | 0·94 |
| Denmark | 7·45 | 13 | 7·68 | 1 | 0·83 | 0·73 | 0·77 | 0·44 | 0·95 |
| Thailand | 7·39 | 14 | 6·1 | 45 | 0·73 | 0·77 | 0·85 | 0·42 | 0·87 |
| United Kingdom | 7·38 | 15 | 6·89 | 18 | 0·76 | 0·76 | 0·73 | 0·53 | 0·91 |
| Austria | 7·38 | 16 | 7·23 | 11 | 0·76 | 0·78 | 0·77 | 0·45 | 0·93 |
| United Arab Emirates | 7·36 | 17 | 6·85 | 20 | 0·75 | 0·8 | 0·82 | 0·45 | 0·91 |
| Costa Rica | 7·34 | 18 | 7·18 | 13 | 0·79 | 0·75 | 0·85 | 0·38 | 0·9 |
| Panama | 7·33 | 19 | 6·68 | 24 | 0·76 | 0·79 | 0·85 | 0·35 | 0·9 |
| Trinidad and Tobago | 7·31 | 20 | 6·28 | 38 | 0·76 | 0·73 | 0·8 | 0·47 | 0·86 |
| Sweden | 7·3 | 21 | 7·36 | 8 | 0·8 | 0·74 | 0·76 | 0·39 | 0·94 |
| Finland | 7·28 | 22 | 7·56 | 2 | 0·77 | 0·74 | 0·78 | 0·4 | 0·94 |
| Paraguay | 7·24 | 23 | 5·57 | 71 | 0·72 | 0·79 | 0·83 | 0·33 | 0·92 |
| Puerto Rico | 7·24 | 24 | 6·82 | 21 | 0·76 | 0·68 | 0·79 | 0·4 | 0·9 |
| Guatemala | 7·23 | 25 | 6·25 | 41 | 0·72 | 0·75 | 0·85 | 0·4 | 0·88 |
| Germany | 7·21 | 26 | 6·72 | 23 | 0·71 | 0·75 | 0·75 | 0·43 | 0·93 |
| Luxembourg | 7·18 | 27 | 7·03 | 16 | 0·74 | 0·75 | 0·75 | 0·4 | 0·93 |
| Malta | 7·17 | 28 | 6·43 | 32 | 0·65 | 0·75 | 0·79 | 0·46 | 0·91 |
| Colombia | 7·16 | 29 | 6·29 | 37 | 0·75 | 0·75 | 0·83 | 0·34 | 0·89 |
| Malaysia | 7·11 | 30 | 5·81 | 56 | 0·7 | 0·79 | 0·79 | 0·39 | 0·85 |
| Bhutan | 7·1 | 31 | 5·2 | 92 | 0·66 | 0·77 | 0·78 | 0·51 | 0·8 |
| Philippines | 7·1 | 32 | 5·28 | 86 | 0·66 | 0·72 | 0·88 | 0·39 | 0·88 |
| Kuwait | 7·02 | 33 | 6·32 | 35 | 0·71 | 0·77 | 0·8 | 0·4 | 0·89 |
| Singapore | 6·99 | 34 | 6·52 | 29 | 0·69 | 0·82 | 0·74 | 0·35 | 0·88 |
| Somaliland | 6·98 | 35 | 4·89 | 108 | 0·69 | 0·78 | 0·79 | 0·4 | 0·81 |
| Sri Lanka | 6·97 | 36 | 4·3 | 141 | 0·61 | 0·66 | 0·8 | 0·48 | 0·85 |
| Dominican Republic | 6·97 | 37 | 5·22 | 90 | 0·67 | 0·71 | 0·82 | 0·39 | 0·86 |
| Taiwan | 6·96 | 38 | 6·29 | 36 | 0·71 | 0·83 | 0·69 | 0·34 | 0·88 |
| Suriname | 6·96 | 39 | 6·27 | 39 | 0·75 | 0·72 | 0·8 | 0·32 | 0·87 |
| Mauritius | 6·94 | 40 | 5·84 | 55 | 0·67 | 0·69 | 0·8 | 0·39 | 0·9 |
| Honduras | 6·93 | 41 | 5·39 | 81 | 0·67 | 0·75 | 0·79 | 0·37 | 0·86 |
| Turkmenistan | 6·91 | 42 | 5·6 | 67 | 0·67 | 0·74 | 0·71 | 0·44 | 0·9 |
| Jamaica | 6·91 | 43 | 5·81 | 58 | 0·71 | 0·73 | 0·8 | 0·39 | 0·8 |
| Nicaragua | 6·89 | 44 | 5·62 | 65 | 0·68 | 0·73 | 0·81 | 0·31 | 0·88 |
| Belgium | 6·85 | 45 | 6·98 | 17 | 0·74 | 0·71 | 0·71 | 0·35 | 0·91 |
| Uruguay | 6·84 | 46 | 6·26 | 40 | 0·74 | 0·73 | 0·72 | 0·29 | 0·89 |
| Mexico | 6·84 | 47 | 6·79 | 22 | 0·75 | 0·76 | 0·76 | 0·28 | 0·85 |
| Kenya | 6·84 | 48 | 4·36 | 138 | 0·62 | 0·74 | 0·8 | 0·44 | 0·76 |
| Chile | 6·84 | 49 | 6·37 | 33 | 0·73 | 0·69 | 0·74 | 0·38 | 0·86 |
| Israel | 6·83 | 50 | 7·22 | 12 | 0·73 | 0·71 | 0·69 | 0·4 | 0·85 |
| Slovenia | 6·82 | 51 | 6 | 48 | 0·61 | 0·73 | 0·75 | 0·37 | 0·92 |
| Ecuador | 6·81 | 52 | 5·76 | 61 | 0·7 | 0·74 | 0·81 | 0·25 | 0·87 |
| Bolivia | 6·8 | 53 | 5·75 | 62 | 0·69 | 0·69 | 0·81 | 0·31 | 0·85 |
| Brazil | 6·79 | 54 | 6·66 | 25 | 0·76 | 0·7 | 0·77 | 0·29 | 0·86 |
| Nigeria | 6·79 | 55 | 4·93 | 106 | 0·65 | 0·76 | 0·79 | 0·42 | 0·74 |
| Myanmar | 6·78 | 56 | 4·39 | 136 | 0·63 | 0·66 | 0·73 | 0·58 | 0·78 |
| Argentina | 6·78 | 57 | 6·34 | 34 | 0·74 | 0·72 | 0·74 | 0·28 | 0·89 |
| Somalia | 6·77 | 58 | 5·15 | 95 | 0·7 | 0·7 | 0·79 | 0·36 | 0·8 |
| Hong Kong | 6·76 | 59 | 5·47 | 77 | 0·6 | 0·81 | 0·64 | 0·44 | 0·86 |
| Cyprus | 6·75 | 60 | 6·04 | 46 | 0·68 | 0·69 | 0·71 | 0·4 | 0·86 |
| El Salvador | 6·74 | 61 | 5·99 | 51 | 0·69 | 0·75 | 0·81 | 0·26 | 0·85 |
| Bahrain | 6·72 | 62 | 5·88 | 53 | 0·66 | 0·72 | 0·8 | 0·41 | 0·9 |
| France | 6·72 | 63 | 6·65 | 26 | 0·71 | 0·72 | 0·7 | 0·31 | 0·91 |
| Venezuela | 6·7 | 64 | 6·11 | 44 | 0·72 | 0·76 | 0·76 | 0·24 | 0·88 |
| Kazakhstan | 6·69 | 65 | 5·78 | 60 | 0·69 | 0·73 | 0·7 | 0·26 | 0·87 |
| Peru | 6·68 | 66 | 5·59 | 68 | 0·69 | 0·7 | 0·8 | 0·29 | 0·82 |
| Libya | 6·67 | 67 | 5·55 | 73 | 0·67 | 0·69 | 0·75 | 0·42 | 0·84 |
| Spain | 6·67 | 68 | 6·49 | 31 | 0·66 | 0·75 | 0·69 | 0·32 | 0·92 |
| Belize | 6·66 | 69 | 6·2 | 43 | 0·69 | 0·75 | 0·81 | 0·35 | 0·79 |
| Kyrgyzstan | 6·66 | 70 | 5·09 | 97 | 0·65 | 0·73 | 0·68 | 0·31 | 0·87 |
| Mongolia | 6·6 | 71 | 4·98 | 102 | 0·66 | 0·77 | 0·58 | 0·38 | 0·83 |
| Gambia | 6·59 | 72 | 4·75 | 116 | 0·65 | 0·56 | 0·79 | 0·51 | 0·77 |
| Estonia | 6·58 | 73 | 5·59 | 69 | 0·66 | 0·71 | 0·68 | 0·25 | 0·91 |
| Poland | 6·58 | 74 | 5·91 | 52 | 0·67 | 0·72 | 0·71 | 0·24 | 0·92 |
| South Africa | 6·55 | 75 | 4·89 | 109 | 0·66 | 0·75 | 0·75 | 0·34 | 0·76 |
| Tajikistan | 6·53 | 76 | 4·98 | 101 | 0·61 | 0·74 | 0·7 | 0·36 | 0·82 |
| Portugal | 6·52 | 77 | 5·37 | 82 | 0·59 | 0·7 | 0·75 | 0·25 | 0·91 |
| China | 6·51 | 78 | 5·01 | 99 | 0·68 | 0·83 | 0·68 | 0·16 | 0·83 |
| Kosovo | 6·51 | 79 | 5·62 | 64 | 0·66 | 0·73 | 0·68 | 0·33 | 0·82 |
| Slovakia | 6·49 | 80 | 6·02 | 47 | 0·68 | 0·71 | 0·65 | 0·25 | 0·9 |
| Ghana | 6·49 | 81 | 4·78 | 113 | 0·63 | 0·71 | 0·79 | 0·4 | 0·73 |
| Northern Cyprus | 6·46 | 82 | 5·69 | 63 | 0·61 | 0·78 | 0·63 | 0·38 | 0·82 |
| Czech Republic | 6·45 | 83 | 6·53 | 28 | 0·69 | 0·72 | 0·68 | 0·23 | 0·87 |
| Italy | 6·43 | 84 | 6·23 | 42 | 0·65 | 0·74 | 0·66 | 0·33 | 0·85 |
| Japan | 6·4 | 85 | 5·99 | 49 | 0·64 | 0·8 | 0·7 | 0·23 | 0·81 |
| Laos | 6·4 | 86 | 4·96 | 104 | 0·69 | 0·71 | 0·77 | 0·35 | 0·76 |
| Namibia | 6·39 | 87 | 4·64 | 121 | 0·6 | 0·76 | 0·79 | 0·3 | 0·76 |
| Zambia | 6·38 | 88 | 4·53 | 124 | 0·61 | 0·69 | 0·78 | 0·38 | 0·71 |
| Vietnam | 6·3 | 89 | 5·33 | 84 | 0·57 | 0·81 | 0·71 | 0·27 | 0·88 |
| South Korea | 6·28 | 90 | 5·88 | 54 | 0·63 | 0·76 | 0·6 | 0·32 | 0·76 |
| Uganda | 6·27 | 91 | 4·28 | 142 | 0·57 | 0·65 | 0·78 | 0·36 | 0·73 |
| Cambodia | 6·26 | 92 | 4·25 | 144 | 0·61 | 0·66 | 0·79 | 0·22 | 0·8 |
| Mali | 6·25 | 93 | 4·25 | 143 | 0·63 | 0·71 | 0·77 | 0·24 | 0·76 |
| Mozambique | 6·25 | 94 | 4·69 | 120 | 0·55 | 0·68 | 0·78 | 0·29 | 0·81 |
| Eswatini | 6·23 | 95 | 4·49 | 127 | 0·66 | 0·63 | 0·74 | 0·31 | 0·77 |
| Senegal | 6·22 | 96 | 4·44 | 132 | 0·62 | 0·68 | 0·78 | 0·29 | 0·74 |
| Mauritania | 6·22 | 97 | 4·46 | 129 | 0·63 | 0·68 | 0·75 | 0·27 | 0·76 |
| Russia | 6·21 | 98 | 5·51 | 76 | 0·63 | 0·69 | 0·63 | 0·21 | 0·86 |
| Latvia | 6·21 | 99 | 5·4 | 79 | 0·63 | 0·69 | 0·61 | 0·22 | 0·89 |
| Botswana | 6·19 | 100 | 3·99 | 157 | 0·58 | 0·69 | 0·77 | 0·3 | 0·76 |
| Liberia | 6·19 | 101 | 4·05 | 152 | 0·53 | 0·61 | 0·78 | 0·46 | 0·69 |
| Nepal | 6·18 | 102 | 4·71 | 118 | 0·6 | 0·69 | 0·65 | 0·35 | 0·74 |
| Cameroon | 6·17 | 103 | 4·57 | 123 | 0·58 | 0·64 | 0·78 | 0·33 | 0·73 |
| Romania | 6·17 | 104 | 5·53 | 75 | 0·62 | 0·64 | 0·69 | 0·24 | 0·83 |
| Lesotho | 6·17 | 105 | 4·01 | 154 | 0·59 | 0·64 | 0·77 | 0·31 | 0·74 |
| Malawi | 6·16 | 106 | 4·06 | 151 | 0·56 | 0·68 | 0·76 | 0·38 | 0·7 |
| Niger | 6·15 | 107 | 4·23 | 145 | 0·6 | 0·64 | 0·8 | 0·25 | 0·78 |
| Zimbabwe | 6·14 | 108 | 3·93 | 160 | 0·56 | 0·71 | 0·72 | 0·28 | 0·74 |
| Lithuania | 6·14 | 109 | 5·81 | 57 | 0·62 | 0·7 | 0·61 | 0·2 | 0·85 |
| Morocco | 6·13 | 110 | 5·04 | 98 | 0·6 | 0·71 | 0·73 | 0·22 | 0·76 |
| Moldova | 6·13 | 111 | 5·6 | 66 | 0·62 | 0·62 | 0·69 | 0·26 | 0·81 |
| Tanzania | 6·13 | 112 | 3·69 | 162 | 0·55 | 0·71 | 0·76 | 0·31 | 0·73 |
| Iran | 6·12 | 113 | 4·82 | 112 | 0·56 | 0·67 | 0·68 | 0·4 | 0·74 |
| Comoros | 6·12 | 114 | 3·86 | 161 | 0·58 | 0·65 | 0·7 | 0·34 | 0·76 |
| Belarus | 6·1 | 115 | 5·57 | 70 | 0·61 | 0·67 | 0·63 | 0·23 | 0·86 |
| Bangladesh | 6·08 | 116 | 4·76 | 115 | 0·6 | 0·69 | 0·67 | 0·28 | 0·78 |
| Rwanda | 6·08 | 117 | 3·68 | 163 | 0·56 | 0·7 | 0·83 | 0·22 | 0·71 |
| Azerbaijan | 6·07 | 118 | 4·94 | 105 | 0·59 | 0·69 | 0·6 | 0·25 | 0·8 |
| Guinea | 6·05 | 119 | 4·09 | 150 | 0·58 | 0·61 | 0·78 | 0·34 | 0·72 |
| Djibouti | 6·04 | 120 | 4·84 | 111 | 0·64 | 0·74 | 0·74 | 0·28 | 0·79 |
| India | 6·03 | 121 | 4·43 | 134 | 0·57 | 0·69 | 0·71 | 0·28 | 0·73 |
| Sierra Leone | 6·01 | 122 | 4·1 | 149 | 0·51 | 0·59 | 0·76 | 0·44 | 0·7 |
| Hungary | 6 | 123 | 5·25 | 88 | 0·61 | 0·66 | 0·6 | 0·22 | 0·85 |
| Sudan | 5·99 | 124 | 4·38 | 137 | 0·56 | 0·66 | 0·66 | 0·38 | 0·78 |
| Ethiopia | 5·98 | 125 | 4·36 | 139 | 0·59 | 0·71 | 0·71 | 0·28 | 0·69 |
| Lebanon | 5·98 | 126 | 4·98 | 103 | 0·53 | 0·71 | 0·65 | 0·31 | 0·81 |
| Burkina Faso | 5·95 | 127 | 4·16 | 147 | 0·56 | 0·66 | 0·72 | 0·26 | 0·75 |
| Angola | 5·93 | 128 | 4·42 | 135 | 0·57 | 0·68 | 0·69 | 0·28 | 0·71 |
| Ivory Coast | 5·92 | 129 | 4·51 | 126 | 0·6 | 0·6 | 0·76 | 0·27 | 0·7 |
| Congo Brazzaville | 5·88 | 130 | 4·49 | 128 | 0·57 | 0·65 | 0·73 | 0·28 | 0·68 |
| North Macedonia | 5·88 | 131 | 4·9 | 107 | 0·57 | 0·67 | 0·65 | 0·25 | 0·79 |
| Ukraine | 5·86 | 132 | 4·74 | 117 | 0·56 | 0·63 | 0·59 | 0·23 | 0·85 |
| Turkey | 5·85 | 133 | 5·26 | 87 | 0·55 | 0·73 | 0·59 | 0·25 | 0·79 |
| Croatia | 5·84 | 134 | 5·55 | 72 | 0·58 | 0·67 | 0·65 | 0·2 | 0·79 |
| Pakistan | 5·83 | 135 | 5·19 | 93 | 0·58 | 0·63 | 0·6 | 0·29 | 0·75 |
| Bulgaria | 5·81 | 136 | 4·44 | 131 | 0·53 | 0·7 | 0·58 | 0·2 | 0·85 |
| Algeria | 5·8 | 137 | 5·4 | 80 | 0·59 | 0·71 | 0·63 | 0·24 | 0·77 |
| Gabon | 5·8 | 138 | 4·43 | 133 | 0·55 | 0·64 | 0·72 | 0·27 | 0·69 |
| Congo Kinshasa | 5·79 | 139 | 4·35 | 140 | 0·58 | 0·7 | 0·68 | 0·21 | 0·7 |
| Greece | 5·76 | 140 | 5·53 | 74 | 0·6 | 0·71 | 0·57 | 0·17 | 0·84 |
| Syria | 5·74 | 141 | 4·1 | 148 | 0·48 | 0·72 | 0·62 | 0·38 | 0·67 |
| Montenegro | 5·73 | 142 | 5·28 | 85 | 0·59 | 0·67 | 0·6 | 0·21 | 0·79 |
| Iraq | 5·73 | 143 | 4·71 | 119 | 0·52 | 0·55 | 0·62 | 0·33 | 0·75 |
| Madagascar | 5·71 | 144 | 3·98 | 158 | 0·56 | 0·65 | 0·66 | 0·21 | 0·77 |
| Palestine | 5·71 | 145 | 4·62 | 122 | 0·55 | 0·63 | 0·65 | 0·2 | 0·8 |
| Albania | 5·7 | 146 | 4·99 | 100 | 0·6 | 0·64 | 0·61 | 0·23 | 0·75 |
| Bosnia Herzegovina | 5·68 | 147 | 5·15 | 96 | 0·56 | 0·64 | 0·59 | 0·25 | 0·76 |
| Egypt | 5·67 | 148 | 4·45 | 130 | 0·52 | 0·66 | 0·63 | 0·24 | 0·8 |
| Serbia | 5·65 | 149 | 5·17 | 94 | 0·56 | 0·65 | 0·6 | 0·18 | 0·79 |
| Tunisia | 5·63 | 150 | 4·78 | 114 | 0·56 | 0·66 | 0·65 | 0·25 | 0·75 |
| Georgia | 5·61 | 151 | 4·23 | 146 | 0·53 | 0·6 | 0·61 | 0·21 | 0·74 |
| Nagorno Karabakh | 5·55 | 152 | 4·86 | 110 | 0·54 | 0·58 | 0·68 | 0·23 | 0·74 |
| Haiti | 5·54 | 153 | 3·96 | 159 | 0·48 | 0·63 | 0·63 | 0·42 | 0·6 |
| Benin | 5·53 | 154 | 4 | 155 | 0·55 | 0·6 | 0·74 | 0·24 | 0·62 |
| Chad | 5·52 | 155 | 4·04 | 153 | 0·53 | 0·61 | 0·67 | 0·26 | 0·68 |
| Yemen | 5·5 | 156 | 4 | 156 | 0·51 | 0·66 | 0·64 | 0·19 | 0·76 |
| Afghanistan | 5·45 | 157 | 3·66 | 164 | 0·47 | 0·69 | 0·62 | 0·31 | 0·63 |
| Armenia | 5·4 | 158 | 4·52 | 125 | 0·51 | 0·57 | 0·61 | 0·23 | 0·75 |
| Central African Republic | 5·33 | 159 | 3·52 | 167 | 0·5 | 0·57 | 0·71 | 0·3 | 0·62 |
| Togo | 5·31 | 160 | 3·56 | 165 | 0·51 | 0·58 | 0·72 | 0·24 | 0·57 |
| Burundi | 5·26 | 161 | 3·55 | 166 | 0·52 | 0·69 | 0·66 | 0·14 | 0·58 |
| South Sudan | 5·22 | 162 | 3·4 | 168 | 0·45 | 0·51 | 0·66 | 0·37 | 0·6 |

Data was pooled across all waves (2009-2019). The composite flourishing and Cantril’s Ladder scores ranged from 0 to 10. Scores for each domain of flourishing ranged from 0 to 1. Higher scores indicate better well-being.

**eTable 3. Gini Index of Composite Flourishing, Cantril’s Ladder Score, and Each Domain of Flourishing Among 162 Countries**

| **Country** | **Flourishing**  **(Gini Index)** | **Flourishing**  **(Rank)** | **Cantril’s Ladder**  **(Gini Index)** | **Cantril’s Ladder**  **(Rank)** | **Domain of Flourishing (Gini Index)** | | | | |
| --- | --- | --- | --- | --- | --- | --- | --- | --- | --- |
|  |  |  |  |  | **Happiness** | **Health** | **Purpose** | **Character** | **Social** |
| Qatar | 0·09 | 1 | 0·17 | 32 | 0·15 | 0·18 | 0·12 | 0·32 | 0·07 |
| Iceland | 0·09 | 2 | 0·12 | 8 | 0·10 | 0·24 | 0·12 | 0·33 | 0·06 |
| Denmark | 0·09 | 3 | 0·11 | 3 | 0·09 | 0·22 | 0·16 | 0·34 | 0·05 |
| New Zealand | 0·09 | 4 | 0·12 | 11 | 0·11 | 0·20 | 0·15 | 0·30 | 0·06 |
| Thailand | 0·10 | 5 | 0·17 | 37 | 0·14 | 0·19 | 0·12 | 0·38 | 0·11 |
| Ireland | 0·10 | 6 | 0·14 | 17 | 0·12 | 0·17 | 0·15 | 0·31 | 0·06 |
| Norway | 0·10 | 7 | 0·11 | 4 | 0·10 | 0·21 | 0·15 | 0·36 | 0·04 |
| United Arab Emirates | 0·10 | 8 | 0·16 | 25 | 0·14 | 0·16 | 0·13 | 0·37 | 0·07 |
| Sweden | 0·10 | 9 | 0·12 | 9 | 0·11 | 0·21 | 0·17 | 0·38 | 0·06 |
| Uzbekistan | 0·10 | 10 | 0·20 | 65 | 0·13 | 0·20 | 0·14 | 0·47 | 0·06 |
| Switzerland | 0·10 | 11 | 0·11 | 5 | 0·12 | 0·19 | 0·14 | 0·37 | 0·05 |
| Netherlands | 0·10 | 12 | 0·09 | 1 | 0·09 | 0·20 | 0·17 | 0·30 | 0·06 |
| Indonesia | 0·10 | 13 | 0·20 | 60 | 0·13 | 0·15 | 0·14 | 0·38 | 0·11 |
| Canada | 0·10 | 14 | 0·12 | 10 | 0·11 | 0·21 | 0·14 | 0·31 | 0·07 |
| Australia | 0·10 | 15 | 0·12 | 12 | 0·12 | 0·21 | 0·16 | 0·29 | 0·06 |
| Luxembourg | 0·10 | 16 | 0·11 | 6 | 0·13 | 0·19 | 0·17 | 0·42 | 0·06 |
| Paraguay | 0·10 | 17 | 0·22 | 82 | 0·15 | 0·19 | 0·13 | 0·56 | 0·08 |
| Bhutan | 0·10 | 18 | 0·12 | 13 | 0·12 | 0·17 | 0·13 | 0·37 | 0·14 |
| Costa Rica | 0·10 | 19 | 0·17 | 30 | 0·13 | 0·21 | 0·12 | 0·47 | 0·09 |
| Panama | 0·10 | 20 | 0·20 | 73 | 0·14 | 0·18 | 0·12 | 0·54 | 0·09 |
| Austria | 0·10 | 21 | 0·13 | 15 | 0·14 | 0·18 | 0·16 | 0·37 | 0·07 |
| Malta | 0·10 | 22 | 0·17 | 31 | 0·19 | 0·20 | 0·13 | 0·34 | 0·08 |
| Finland | 0·10 | 23 | 0·10 | 2 | 0·13 | 0·21 | 0·16 | 0·43 | 0·05 |
| Philippines | 0·11 | 24 | 0·26 | 127 | 0·18 | 0·22 | 0·10 | 0·47 | 0·10 |
| Turkmenistan | 0·11 | 25 | 0·17 | 36 | 0·16 | 0·20 | 0·18 | 0·40 | 0·08 |
| Colombia | 0·11 | 26 | 0·23 | 92 | 0·15 | 0·21 | 0·13 | 0·48 | 0·10 |
| Kuwait | 0·11 | 27 | 0·17 | 35 | 0·15 | 0·18 | 0·13 | 0·38 | 0·09 |
| Syria | 0·11 | 28 | 0·34 | 159 | 0·27 | 0·19 | 0·21 | 0·37 | 0·23 |
| United States | 0·11 | 29 | 0·14 | 20 | 0·12 | 0·22 | 0·15 | 0·29 | 0·09 |
| Laos | 0·11 | 30 | 0·18 | 39 | 0·12 | 0·23 | 0·13 | 0·48 | 0·17 |
| United Kingdom | 0·11 | 31 | 0·14 | 19 | 0·13 | 0·20 | 0·18 | 0·31 | 0·08 |
| Germany | 0·11 | 32 | 0·15 | 21 | 0·16 | 0·21 | 0·17 | 0·38 | 0·06 |
| Guatemala | 0·11 | 33 | 0·24 | 100 | 0·17 | 0·21 | 0·12 | 0·49 | 0·11 |
| Taiwan | 0·11 | 34 | 0·15 | 24 | 0·14 | 0·14 | 0·21 | 0·48 | 0·10 |
| Malaysia | 0·11 | 35 | 0·17 | 33 | 0·14 | 0·17 | 0·15 | 0·49 | 0·12 |
| Trinidad and Tobago | 0·11 | 36 | 0·20 | 66 | 0·14 | 0·22 | 0·14 | 0·41 | 0·12 |
| Sri Lanka | 0·11 | 37 | 0·24 | 103 | 0·18 | 0·25 | 0·14 | 0·39 | 0·12 |
| Singapore | 0·11 | 38 | 0·13 | 14 | 0·15 | 0·14 | 0·17 | 0·49 | 0·10 |
| Libya | 0·11 | 39 | 0·24 | 106 | 0·18 | 0·21 | 0·15 | 0·35 | 0·12 |
| Honduras | 0·12 | 40 | 0·30 | 149 | 0·20 | 0·21 | 0·15 | 0·52 | 0·11 |
| France | 0·12 | 41 | 0·14 | 18 | 0·15 | 0·21 | 0·20 | 0·50 | 0·08 |
| Mauritius | 0·12 | 42 | 0·20 | 62 | 0·18 | 0·24 | 0·13 | 0·48 | 0·09 |
| Jamaica | 0·12 | 43 | 0·25 | 114 | 0·17 | 0·21 | 0·14 | 0·44 | 0·15 |
| Spain | 0·12 | 44 | 0·15 | 22 | 0·18 | 0·20 | 0·20 | 0·46 | 0·07 |
| Argentina | 0·12 | 45 | 0·18 | 45 | 0·14 | 0·23 | 0·18 | 0·55 | 0·10 |
| Belize | 0·12 | 46 | 0·20 | 68 | 0·17 | 0·20 | 0·14 | 0·51 | 0·16 |
| Nicaragua | 0·12 | 47 | 0·28 | 141 | 0·19 | 0·22 | 0·14 | 0·57 | 0·10 |
| Uruguay | 0·12 | 48 | 0·20 | 69 | 0·15 | 0·23 | 0·19 | 0·55 | 0·10 |
| Mexico | 0·12 | 49 | 0·18 | 41 | 0·15 | 0·20 | 0·17 | 0·57 | 0·13 |
| Brazil | 0·12 | 50 | 0·20 | 72 | 0·15 | 0·25 | 0·16 | 0·55 | 0·12 |
| Northern Cyprus | 0·12 | 51 | 0·20 | 70 | 0·21 | 0·16 | 0·19 | 0·43 | 0·14 |
| Bolivia | 0·12 | 52 | 0·19 | 59 | 0·17 | 0·25 | 0·14 | 0·54 | 0·12 |
| El Salvador | 0·12 | 53 | 0·24 | 98 | 0·18 | 0·21 | 0·14 | 0·60 | 0·12 |
| Ecuador | 0·12 | 54 | 0·23 | 97 | 0·17 | 0·23 | 0·14 | 0·62 | 0·12 |
| Belgium | 0·12 | 55 | 0·11 | 7 | 0·13 | 0·23 | 0·19 | 0·48 | 0·08 |
| Dominican Republic | 0·12 | 56 | 0·35 | 162 | 0·22 | 0·24 | 0·13 | 0·47 | 0·11 |
| Somalia | 0·12 | 57 | 0·26 | 128 | 0·15 | 0·23 | 0·12 | 0·45 | 0·15 |
| Myanmar | 0·12 | 58 | 0·24 | 105 | 0·18 | 0·26 | 0·15 | 0·28 | 0·16 |
| Nigeria | 0·12 | 59 | 0·25 | 117 | 0·18 | 0·18 | 0·14 | 0·42 | 0·19 |
| Cyprus | 0·12 | 60 | 0·20 | 64 | 0·17 | 0·23 | 0·18 | 0·43 | 0·11 |
| Bahrain | 0·12 | 61 | 0·20 | 63 | 0·19 | 0·22 | 0·13 | 0·45 | 0·08 |
| Vietnam | 0·12 | 62 | 0·16 | 27 | 0·21 | 0·16 | 0·17 | 0·56 | 0·10 |
| Slovenia | 0·12 | 63 | 0·19 | 49 | 0·22 | 0·22 | 0·17 | 0·43 | 0·07 |
| Suriname | 0·12 | 64 | 0·16 | 26 | 0·12 | 0·22 | 0·13 | 0·53 | 0·11 |
| China | 0·12 | 65 | 0·22 | 86 | 0·15 | 0·16 | 0·21 | 0·69 | 0·13 |
| Portugal | 0·12 | 66 | 0·22 | 79 | 0·23 | 0·23 | 0·17 | 0·55 | 0·08 |
| Chile | 0·12 | 67 | 0·19 | 54 | 0·16 | 0·26 | 0·18 | 0·45 | 0·11 |
| Hong Kong | 0·12 | 68 | 0·18 | 42 | 0·20 | 0·15 | 0·22 | 0·37 | 0·11 |
| Peru | 0·12 | 69 | 0·22 | 89 | 0·17 | 0·24 | 0·15 | 0·56 | 0·14 |
| Venezuela | 0·12 | 70 | 0·25 | 112 | 0·18 | 0·21 | 0·17 | 0·62 | 0·10 |
| Poland | 0·12 | 71 | 0·18 | 40 | 0·18 | 0·23 | 0·18 | 0·61 | 0·08 |
| Kazakhstan | 0·12 | 72 | 0·18 | 43 | 0·16 | 0·22 | 0·20 | 0·61 | 0·11 |
| Italy | 0·13 | 73 | 0·15 | 23 | 0·18 | 0·20 | 0·22 | 0·49 | 0·12 |
| Puerto Rico | 0·13 | 74 | 0·22 | 85 | 0·17 | 0·27 | 0·16 | 0·46 | 0·08 |
| Mongolia | 0·13 | 75 | 0·20 | 61 | 0·18 | 0·20 | 0·24 | 0·49 | 0·13 |
| Ghana | 0·13 | 76 | 0·26 | 122 | 0·19 | 0·21 | 0·14 | 0·45 | 0·20 |
| South Africa | 0·13 | 77 | 0·25 | 118 | 0·17 | 0·19 | 0·17 | 0·46 | 0·17 |
| Gambia | 0·13 | 78 | 0·36 | 164 | 0·21 | 0·32 | 0·15 | 0·36 | 0·18 |
| Senegal | 0·13 | 79 | 0·21 | 76 | 0·17 | 0·23 | 0·15 | 0·49 | 0·18 |
| Namibia | 0·13 | 80 | 0·29 | 143 | 0·21 | 0·19 | 0·15 | 0·49 | 0·18 |
| Kyrgyzstan | 0·13 | 81 | 0·19 | 53 | 0·18 | 0·24 | 0·18 | 0·55 | 0·12 |
| Kenya | 0·13 | 82 | 0·25 | 115 | 0·18 | 0·19 | 0·14 | 0·42 | 0·19 |
| Japan | 0·13 | 83 | 0·18 | 38 | 0·19 | 0·16 | 0·20 | 0·62 | 0·15 |
| Mozambique | 0·13 | 84 | 0·30 | 150 | 0·24 | 0·23 | 0·15 | 0·54 | 0·14 |
| Cambodia | 0·13 | 85 | 0·28 | 140 | 0·20 | 0·28 | 0·13 | 0·61 | 0·15 |
| Cameroon | 0·13 | 86 | 0·26 | 126 | 0·22 | 0·26 | 0·15 | 0·46 | 0·20 |
| Israel | 0·13 | 87 | 0·13 | 16 | 0·16 | 0·22 | 0·21 | 0·46 | 0·12 |
| Mali | 0·13 | 88 | 0·26 | 123 | 0·18 | 0·23 | 0·16 | 0·55 | 0·18 |
| Mauritania | 0·13 | 89 | 0·22 | 87 | 0·17 | 0·23 | 0·17 | 0·54 | 0·18 |
| Niger | 0·13 | 90 | 0·26 | 120 | 0·18 | 0·27 | 0·14 | 0·53 | 0·17 |
| Eswatini | 0·13 | 91 | 0·30 | 146 | 0·17 | 0·27 | 0·16 | 0·49 | 0·17 |
| Zambia | 0·13 | 92 | 0·29 | 145 | 0·20 | 0·23 | 0·14 | 0·42 | 0·20 |
| Morocco | 0·13 | 93 | 0·22 | 90 | 0·22 | 0·25 | 0·18 | 0·52 | 0·18 |
| Tajikistan | 0·13 | 94 | 0·19 | 48 | 0·20 | 0·22 | 0·19 | 0·49 | 0·14 |
| Somaliland | 0·14 | 95 | 0·22 | 81 | 0·16 | 0·17 | 0·15 | 0·45 | 0·16 |
| Lesotho | 0·14 | 96 | 0·41 | 167 | 0·23 | 0·29 | 0·15 | 0·43 | 0·18 |
| Guinea | 0·14 | 97 | 0·31 | 154 | 0·21 | 0·28 | 0·16 | 0·48 | 0·20 |
| Burkina Faso | 0·14 | 98 | 0·25 | 110 | 0·21 | 0·25 | 0·18 | 0·56 | 0·18 |
| Botswana | 0·14 | 99 | 0·32 | 156 | 0·22 | 0·24 | 0·15 | 0·47 | 0·17 |
| Kosovo | 0·14 | 100 | 0·22 | 88 | 0·19 | 0·22 | 0·19 | 0·52 | 0·15 |
| Bangladesh | 0·14 | 101 | 0·25 | 107 | 0·22 | 0·25 | 0·14 | 0·53 | 0·16 |
| Czech Republic | 0·14 | 102 | 0·16 | 28 | 0·18 | 0·24 | 0·22 | 0·64 | 0·11 |
| Nepal | 0·14 | 103 | 0·26 | 125 | 0·21 | 0·25 | 0·17 | 0·54 | 0·19 |
| Comoros | 0·14 | 104 | 0·27 | 136 | 0·20 | 0·27 | 0·19 | 0·51 | 0·18 |
| Ivory Coast | 0·14 | 105 | 0·29 | 142 | 0·20 | 0·29 | 0·16 | 0·50 | 0·21 |
| Estonia | 0·14 | 106 | 0·19 | 55 | 0·19 | 0·26 | 0·23 | 0·63 | 0·08 |
| Madagascar | 0·14 | 107 | 0·24 | 102 | 0·20 | 0·25 | 0·20 | 0·66 | 0·17 |
| Malawi | 0·14 | 108 | 0·37 | 165 | 0·24 | 0·24 | 0·15 | 0·45 | 0·21 |
| Uganda | 0·14 | 109 | 0·29 | 144 | 0·22 | 0·26 | 0·15 | 0·45 | 0·20 |
| Latvia | 0·14 | 110 | 0·19 | 56 | 0·20 | 0·25 | 0·26 | 0·64 | 0·10 |
| Congo Kinshasa | 0·14 | 111 | 0·21 | 78 | 0·19 | 0·22 | 0·19 | 0·60 | 0·20 |
| Russia | 0·14 | 112 | 0·21 | 75 | 0·21 | 0·25 | 0·24 | 0·65 | 0·11 |
| Zimbabwe | 0·14 | 113 | 0·31 | 152 | 0·24 | 0·23 | 0·18 | 0·52 | 0·19 |
| Turkey | 0·15 | 114 | 0·23 | 93 | 0·25 | 0·20 | 0·23 | 0·56 | 0·17 |
| Slovakia | 0·15 | 115 | 0·18 | 46 | 0·19 | 0·26 | 0·24 | 0·62 | 0·09 |
| Tanzania | 0·15 | 116 | 0·32 | 158 | 0·22 | 0·23 | 0·16 | 0·52 | 0·19 |
| Liberia | 0·15 | 117 | 0·40 | 166 | 0·26 | 0·28 | 0·15 | 0·33 | 0·22 |
| Rwanda | 0·15 | 118 | 0·28 | 137 | 0·20 | 0·24 | 0·12 | 0·64 | 0·21 |
| Greece | 0·15 | 119 | 0·23 | 91 | 0·24 | 0·23 | 0·24 | 0·65 | 0·13 |
| Iran | 0·15 | 120 | 0·27 | 132 | 0·26 | 0·25 | 0·19 | 0·42 | 0·18 |
| Azerbaijan | 0·15 | 121 | 0·19 | 58 | 0·22 | 0·25 | 0·24 | 0·61 | 0·16 |
| Congo Brazzaville | 0·15 | 122 | 0·34 | 160 | 0·23 | 0·27 | 0·17 | 0·49 | 0·23 |
| Gabon | 0·15 | 123 | 0·26 | 129 | 0·23 | 0·26 | 0·18 | 0·48 | 0·21 |
| India | 0·15 | 124 | 0·27 | 135 | 0·22 | 0·23 | 0·18 | 0·60 | 0·20 |
| Ethiopia | 0·15 | 125 | 0·25 | 108 | 0·21 | 0·20 | 0·17 | 0·55 | 0·23 |
| Angola | 0·15 | 126 | 0·30 | 147 | 0·23 | 0·22 | 0·18 | 0·61 | 0·20 |
| Sudan | 0·15 | 127 | 0·22 | 84 | 0·21 | 0·25 | 0·20 | 0·39 | 0·16 |
| Pakistan | 0·15 | 128 | 0·25 | 116 | 0·24 | 0·27 | 0·21 | 0·57 | 0·17 |
| Djibouti | 0·15 | 129 | 0·21 | 74 | 0·18 | 0·20 | 0·18 | 0·55 | 0·17 |
| Belarus | 0·16 | 130 | 0·19 | 50 | 0·22 | 0·27 | 0·23 | 0·64 | 0·12 |
| Hungary | 0·16 | 131 | 0·22 | 80 | 0·21 | 0·29 | 0·26 | 0·64 | 0·12 |
| Sierra Leone | 0·16 | 132 | 0·36 | 163 | 0·25 | 0·30 | 0·16 | 0·38 | 0·21 |
| South Korea | 0·16 | 133 | 0·20 | 71 | 0·22 | 0·20 | 0·25 | 0·53 | 0·19 |
| Romania | 0·16 | 134 | 0·23 | 96 | 0·24 | 0·33 | 0·19 | 0·61 | 0·14 |
| Egypt | 0·16 | 135 | 0·27 | 134 | 0·27 | 0·28 | 0·19 | 0·55 | 0·15 |
| Iraq | 0·16 | 136 | 0·26 | 121 | 0·25 | 0·32 | 0·22 | 0·44 | 0·18 |
| Moldova | 0·16 | 137 | 0·20 | 67 | 0·22 | 0·32 | 0·21 | 0·60 | 0·15 |
| Chad | 0·16 | 138 | 0·28 | 139 | 0·22 | 0·28 | 0·19 | 0·56 | 0·23 |
| Lebanon | 0·16 | 139 | 0·25 | 113 | 0·28 | 0·23 | 0·21 | 0·52 | 0·15 |
| Lithuania | 0·16 | 140 | 0·19 | 57 | 0·22 | 0·25 | 0·25 | 0·65 | 0·13 |
| Benin | 0·16 | 141 | 0·31 | 153 | 0·22 | 0·29 | 0·17 | 0·60 | 0·26 |
| Algeria | 0·16 | 142 | 0·19 | 51 | 0·21 | 0·23 | 0·21 | 0·59 | 0·17 |
| Central African Republic | 0·16 | 143 | 0·32 | 155 | 0·25 | 0·32 | 0·17 | 0·54 | 0·24 |
| Ukraine | 0·16 | 144 | 0·24 | 101 | 0·24 | 0·30 | 0·25 | 0·63 | 0·13 |
| Tunisia | 0·16 | 145 | 0·23 | 95 | 0·24 | 0·26 | 0·21 | 0·52 | 0·19 |
| Palestine | 0·17 | 146 | 0·27 | 131 | 0·26 | 0·30 | 0·21 | 0·62 | 0·15 |
| North Macedonia | 0·17 | 147 | 0·25 | 111 | 0·25 | 0·26 | 0·21 | 0·61 | 0·16 |
| Croatia | 0·17 | 148 | 0·19 | 52 | 0·23 | 0·25 | 0·22 | 0·66 | 0·16 |
| Nagorno Karabakh | 0·17 | 149 | 0·18 | 44 | 0·24 | 0·32 | 0·18 | 0·53 | 0·18 |
| Togo | 0·17 | 150 | 0·34 | 161 | 0·24 | 0·29 | 0·18 | 0·60 | 0·29 |
| Yemen | 0·17 | 151 | 0·32 | 157 | 0·29 | 0·27 | 0·19 | 0·60 | 0·18 |
| Bulgaria | 0·17 | 152 | 0·26 | 119 | 0·28 | 0·27 | 0·27 | 0·65 | 0·12 |
| Montenegro | 0·17 | 153 | 0·23 | 94 | 0·24 | 0·25 | 0·25 | 0·62 | 0·16 |
| Albania | 0·17 | 154 | 0·26 | 124 | 0·23 | 0·30 | 0·23 | 0·61 | 0·18 |
| Burundi | 0·17 | 155 | 0·31 | 151 | 0·25 | 0·24 | 0·21 | 0·75 | 0·29 |
| Haiti | 0·18 | 156 | 0·30 | 148 | 0·27 | 0·29 | 0·23 | 0·44 | 0·28 |
| Bosnia Herzegovina | 0·18 | 157 | 0·24 | 99 | 0·25 | 0·28 | 0·25 | 0·58 | 0·18 |
| Serbia | 0·18 | 158 | 0·25 | 109 | 0·27 | 0·28 | 0·26 | 0·68 | 0·17 |
| Georgia | 0·19 | 159 | 0·27 | 130 | 0·28 | 0·35 | 0·22 | 0·62 | 0·18 |
| South Sudan | 0·19 | 160 | 0·48 | 168 | 0·33 | 0·35 | 0·21 | 0·46 | 0·27 |
| Afghanistan | 0·19 | 161 | 0·28 | 138 | 0·27 | 0·24 | 0·21 | 0·51 | 0·26 |
| Armenia | 0·19 | 162 | 0·27 | 133 | 0·30 | 0·36 | 0·24 | 0·58 | 0·18 |

Data was pooled across all waves (2009-2019). Higher rank for Gini Index indicates less inequality in wellbeing.


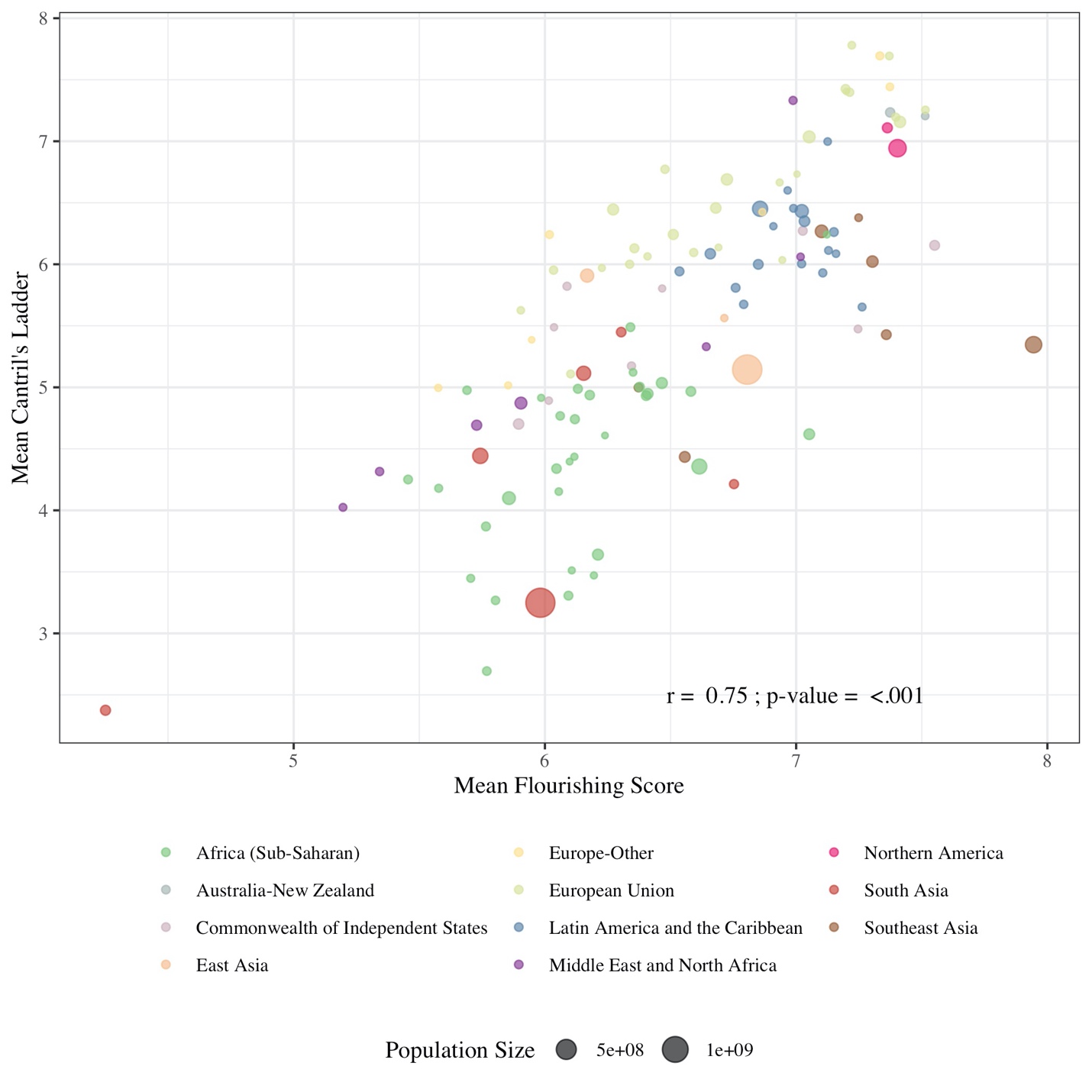


**eFigure 1.** Correlation Between Country-specific Mean Flourishing and Mean Cantril’s Ladder in 2019.

Pearson’s correlation coefficients were calculated. Points were colored by regions· Size of each point represents the country’s population size.

**eFigure 2.** Trajectories of Mean Score for Composite Flourishing and Each Domain of Flourishing from 2009 to 2019 by Region.

**eFigure 3.** Trajectories of Gini Index for Composite Flourishing and Each Domain of Flourishing from 2009 to 2019 by Region.

**
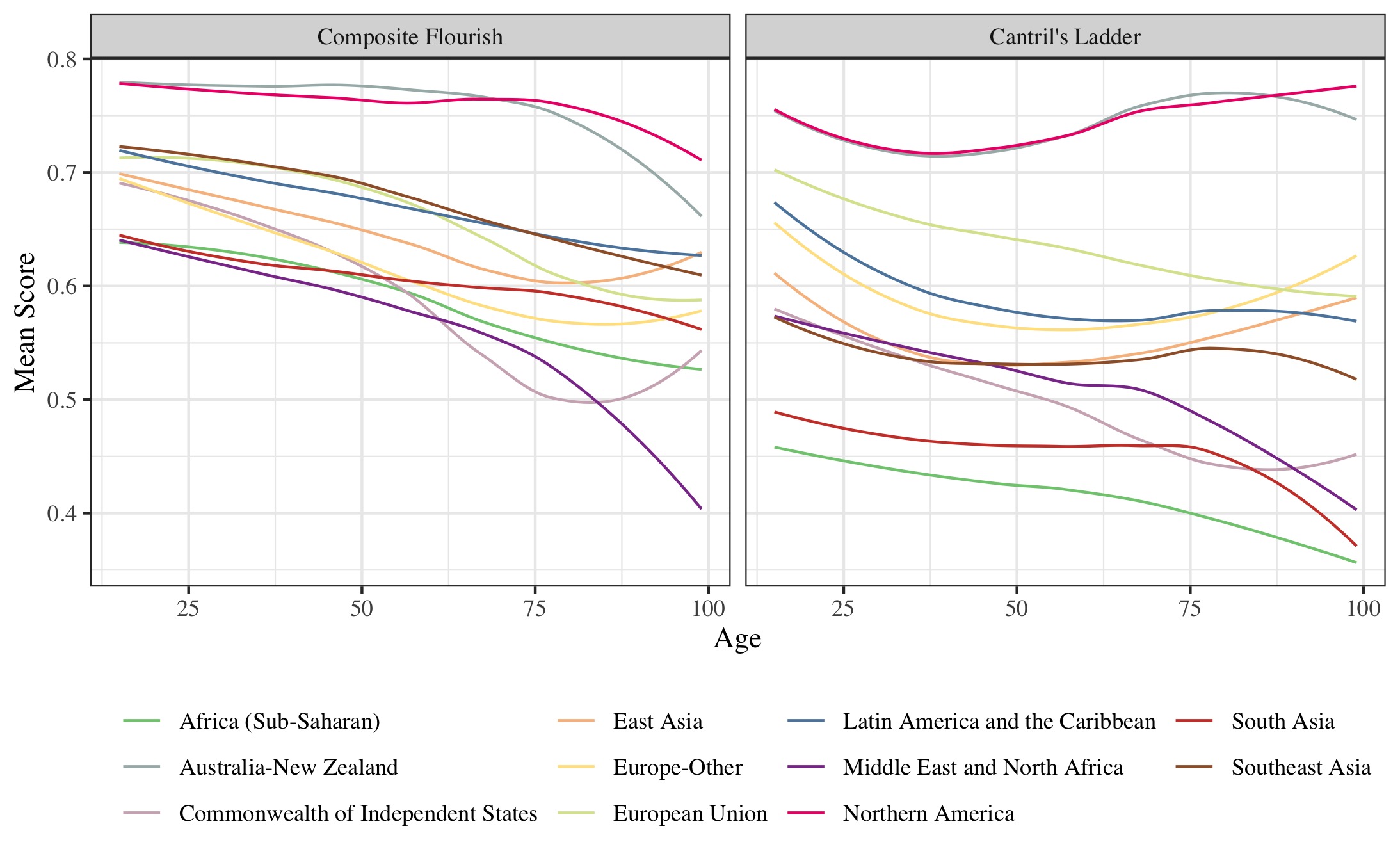
**

**eFigure 4.** Associations of Composite Flourishing/Cantril’s Ladder with Age. Data was pooled across years·
